# Methylglyoxal-Induced Modifications in Human Triosephosphate Isomerase: Structural and Functional Repercussions of Specific Mutations

**DOI:** 10.3390/molecules29215047

**Published:** 2024-10-25

**Authors:** Ignacio de la Mora-de la Mora, Itzhel García-Torres, Luis Antonio Flores-López, Gabriel López-Velázquez, Gloria Hernández-Alcántara, Saúl Gómez-Manzo, Sergio Enríquez-Flores

**Affiliations:** 1 Laboratorio de Biomoléculas y Salud Infantil, Instituto Nacional de Pediatría, Secretaría de Salud, Mexico City 04530, Mexico; itzheltorres@hotmail.com (I.G.-T.); luisbiolexp@gmail.com (L.A.F.-L.); glv_1999@ciencias.unam.mx (G.L.-V.); 2 Consejo Nacional de Humanidades, Ciencias y Tecnologías (CONAHCYT)-Instituto Nacional de Pediatría, Secretaría de Salud, Mexico City 04530, Mexico; 3 Departamento de Bioquímica, Facultad de Medicina, Universidad Nacional Autónoma de México (UNAM), Mexico City 04510, Mexico; ghernandez@bq.unam.mx; 4 Laboratorio de Bioquímica Genética, Instituto Nacional de Pediatría, Secretaría de Salud, Mexico City 04530, Mexico; saulmanzo@ciencias.unam.mx

**Keywords:** spectroscopic analysis, protein structure–function relationship, molecular dysfunction, conformational changes, molecular wear, catalytic aging, enzymatic rescue, molecular docking

## Abstract

Triosephosphate isomerase (TPI) dysfunction is a critical factor in diverse pathological conditions. Deficiencies in TPI lead to the accumulation of toxic methylglyoxal (MGO), which induces non-enzymatic post-translational modifications, thus compromising protein stability and leading to misfolding. This study investigates how specific TPI mutations (E104D, N16D, and C217K) affect the enzyme’s structural stability when exposed to its substrate glyceraldehyde 3-phosphate (G3P) and MGO. We employed circular dichroism, intrinsic fluorescence, native gel electrophoresis, and Western blotting to assess the structural alterations and aggregation propensity of these TPI mutants. Our findings indicate that these mutations markedly increase TPI’s susceptibility to MGO-induced damage, leading to accelerated loss of enzymatic activity and enhanced protein aggregation. Additionally, we observed the formation of MGO-induced adducts, such as argpyrimidine (ARGp), that contribute to enzyme inactivation and aggregation. Importantly, the application of MGO-scavenging molecules partially mitigated these deleterious effects, highlighting potential therapeutic strategies to counteract MGO-induced damage in TPI-related disorders.

## 1. Introduction

Metabolic alterations in the glycolytic pathway, caused by defects in triosephosphate isomerase (TPI), result in the accumulation of toxic intermediates such as methylglyoxal (MGO). This is mainly due to the fact that TPI catalyzes the reversible conversion of dihydroxyacetone phosphate (DHAP) and glyceraldehyde 3-phosphate (G3P), preventing their buildup. When TPI activity is deficient, these substrates accumulate and degrade into MGO, a reactive dicarbonyl with toxic potential [1,2,3].

Increased levels of MGO have been shown to promote non-enzymatic post-translational modifications (NE/PTM), which compromise proteins’ stability and lead to misfolding, as previously reported for TPI from *Drosophila* [4]. MGO primarily modifies proteins by forming adducts with arginine (Arg) residues, leading to hydroimidazolone adducts (MGO-H) [5]. It can also target lysine (Lys) residues, generating reactive oxygen species such as superoxide [6], which increase intracellular oxidative stress [7]. Additionally, MGO also binds nucleophilic molecules, including thiols such as glutathione (GSH) and cysteine (Cys) residues in proteins, forming hemithioacetals [8]. Through these mechanisms, MGO contributes to a plethora of chronic degenerative diseases, including arterial hypertension, atherosclerosis, diabetes mellitus, cancer [9], Parkinson’s disease [10], and Alzheimer’s disease (AD) [11], among others. Many of these diseases are characterized by the accumulation of proteins with impaired metabolic functions due to NE/PTMs, such as albumin [12,13], hemoglobin [14], HSP-27 [15], G3-PDH [16], α-synuclein [17], and TPI [18], gradually leading to a feedback link that exacerbates disease progression.

Although MGO is toxic, it exhibits a paradoxical effect in cancer and metabolic syndrome, where increased glycolytic activity (30- to 200-fold compared to normal cells) supports cell proliferation, exerting a hormetic effect [19]. Despite MGO’s detrimental potential, it is detoxified under normal conditions via both GSH-dependent and GSH-independent pathways [20], ensuring cellular homeostasis. However, glycolytic enzyme defects, such as those involving TPI, disrupt these mechanisms, leading to toxic intermediate accumulation.

TPI is essential for glycolysis by catalyzing the interconversion between DHAP and G3P, driving ATP production and other metabolites such as NADH. The enzyme also prevents the accumulation of these trioses, which would otherwise lead to its accumulation and subsequent degradation into toxic MGO.

Some genetic mutations of the human TPI1 gene (located on chromosome 12p13.31) have been shown to be associated with metabolic disorders, including a rare autosomal recessive TPI deficiency that presents with severe neurological symptoms and hemolytic anemia [21]. Structural mutations such as E104D (where glutamic acid [E] is replaced by aspartic acid [D] at position 104) impair enzyme folding and stability, thereby disrupting glycolysis and promoting the accumulation of toxic intermediates [22]. Thus, disruptions in TPI structure or function due to autosomal mutations, protein aging, or post-translational modifications (PTMs) can lead to several metabolic disorders [21,22,23,24]. Aging and pathological conditions such as cancer are associated with deamidation of Asparagine at position 16 (N16), a residue located at the enzyme’s dimer interface. The spontaneous conversion of N16 to aspartic acid (N16D) destabilizes the dimer interface and promotes NE/PTMs, further exacerbating MGO accumulation and glycolytic dysfunction [25,26]. Moreover, modifications of cysteine 217 (Cys217) through processes like *S*-nitrosylation impair enzyme activity by interfering with the efficient interconversion of DHAP and G3P [27]. These modifications highlight the importance of TPI regulation and demonstrate how minor structural alterations can have profound pathological effects.

The interplay between TPI mutations and NE/PTMs can lead to pathological consequences. Under certain conditions, TPI modifications such as nitrotyrosination impair glycolytic flux, increase MGO levels, and promote glycation [23]. These processes contribute to the formation of advanced glycation end-products (AGEs) [28], which exacerbate disease progression, as observed in Alzheimer’s disease (AD) [23]. Therefore, it is important to highlight that glycolytic failure leads to the accumulation of harmful adducts and dicarbonyl stress, thus promoting the formation of AGEs [29]. AGEs, with diverse chemical structures, could be associated with different pathologies, complicating, in part, their quantification [29]. These AGEs irreversibly modify Lys and Arg residues [30], allowing for the classification of non-fluorescent types (e.g., *N*-ε-(carboxyethyl)lysine [CEL]) and carboxymethyl-lysine [CML]) and fluorescent types (e.g., pentosidine, MGO-lysine dimer [MOLD], and ARGp) [31].

Given the critical role of TPI in glycolysis and its susceptibility to mutations and NE/PTMs, this study investigates the structural and functional alterations caused by single mutations (E104D, N16D, and C217K) and their relationship with MGO formation. These mutations target key residues: E104D impairs protein stability, N16D disrupts the dimer interface, and C217K involves the modification of a cysteine residue linked to regulatory processes. By examining these variants in the presence of G3P and MGO, we aim to elucidate the mechanisms through which these mutations contribute to MGO accumulation and metabolic dysfunction.

## 2. Results

### 2.1. Kinetic Analysis

Kinetic parameters were determined to understand the functional consequences of recombinant HsTPI mutants (N16D, E104D, and C217K) relative to the WT enzyme. As shown in Table 1, each mutation distinctly affected enzyme behavior. The C217K mutant’s significant increase in *V_max_* was approximately 3.28-fold higher than the WT, suggesting enhanced maximal catalytic activity. However, this increase was coupled with a 3.82-fold elevation in *K*_m_, reflecting a substantial decrease in substrate affinity.

While the catalytic rate (*k*_cat_) of C217K was notably higher (at least 2.2 times that of WT and E104D), the reduced substrate affinity led to catalytic efficiency (*k*_cat_/*K*_m_) nearly equivalent to that of WT and E104D.

In contrast, the N16D mutant exhibited a pronounced decline in catalytic efficiency, primarily due to a significant decrease in both *V_max_* and *k*_cat_, as well as poor substrate affinity, indicating that the N16D is the most detrimental mutation in terms of enzymatic activity. The N16D mutation, which involves the substitution of an Asn (neutral) residue by Asp (negatively charged), disrupts local hydrogen bonding networks and electrostatic interactions important for binding between protein monomers to form the dimer. These structural changes are known to destabilize the active site [22], leading to poor substrate affinity and a possible change in the enzyme conformation, either by locking it in a nonproductive state or by exposing non-catalytic binding sites that induce substrate inhibition. Thus, these findings align with previous studies [22] but also emphasize the differential impact of specific mutations on the catalytic properties of HsTPI. The C217K mutation, while increasing overall activity, compromises substrate affinity, leading to a net neutral effect on catalytic efficiency. Conversely, the N16D mutation severely impairs the enzyme’s catalytic function, underscoring its potential pathological relevance.

### 2.2. Cysteine Accessibility Assay Revealed a Compact Structure for the C217K Mutant and Structural Relaxation in the E104D and N16D Mutants

**Figure 1:**
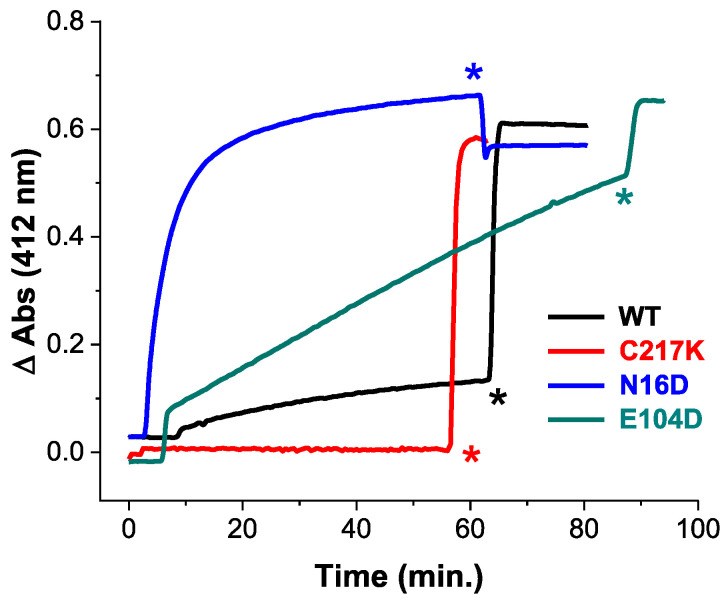
Cysteine quantification under native conditions of HsTPI WT and mutants. Initial substrate-free enzyme permeability by DTNB derivatization of free Cys under native conditions in the WT-HsTPI WT and the C217K, N16D, and E104D mutants. Samples containing 200 µg of recombinant protein in TE buffer at pH 7.4 were exposed to 4 mM DTNB, and spectrophotometric absorbance readings were taken at 412 nm and monitored every 30 sec for 60 min at 25 °C. SDS was then added to completely denature the enzymes. HsTPI-WT (black). Mutants: E104D (green), C217K (red), N16D (blue). These results are representative of qualitatively identical duplicate experiments with a difference of less than 5%. Asterisks indicate the time of addition of 20% SDS.

To elucidate the impact of these mutations, we employed Cys derivatization by Ellman’s reagent, or DTNB (5,5′-dithiobis-(2-nitrobenzoic acid). This reagent is mainly employed in the derivatization of thiols. The principle of the reaction is based on a thiol-exchange reaction forming a thiol-5-thio-2-nitrobenzoic acid adduct and a release of one equivalent of 5-thio-2-nitrobenzoic acid (TNB), followed by absorbance at 412 nm. We measured structural alterations in recombinant TPIs, using the accessibility of free cysteine (Cys) residues by DTNB under native conditions. As shown in Figure 1, differential Cys accessibility to DTNB among HsTPI mutants compared to the WT reflects variations in protein compaction.

The C217K mutant demonstrated no derivatization Cys residues within the first hour, strongly suggesting a highly compact native conformation that restricts DTNB access. Whereas the WT enzyme showed derivatization of approximately one Cys per subunit, suggesting moderate exposure to Cys residues. The E104D mutant demonstrated a significantly higher and progressive rate of Cys derivatization, with four Cys per subunit derivatized within the first hour. This indicates great accessibility to the DTNB ligand, likely due to the substitution of Glu with Asp at position 104, which disrupts the native structure. Notably, the N16D mutant showed ever faster Cys derivatization, with four Cys per subunit modified within 20 min (Supplementary Table S1). This rapid derivatization suggests extensive accessibility, likely resulting from the introduction of Asp at position 16, which is near the protein’s interfacial region [22]. Following the addition of a denaturing agent, all enzymes except C217K showed quantification of five Cys per subunit (Supplementary Table S1). However, the C217K mutant showed only four Cys per subunit, aligning with the expected number of Cys residues that should be titrated.

The above results underline the differential impacts of mutations on HsTPI structure, with N16D and E104D leading to more pronounced structural accessibility to DTNB compared to the C217K mutant.

### 2.3. G3P Binding Induces Distinct Shifts in Electrophoretic Mobility Among HsTPI Mutants

**Figure 2:**
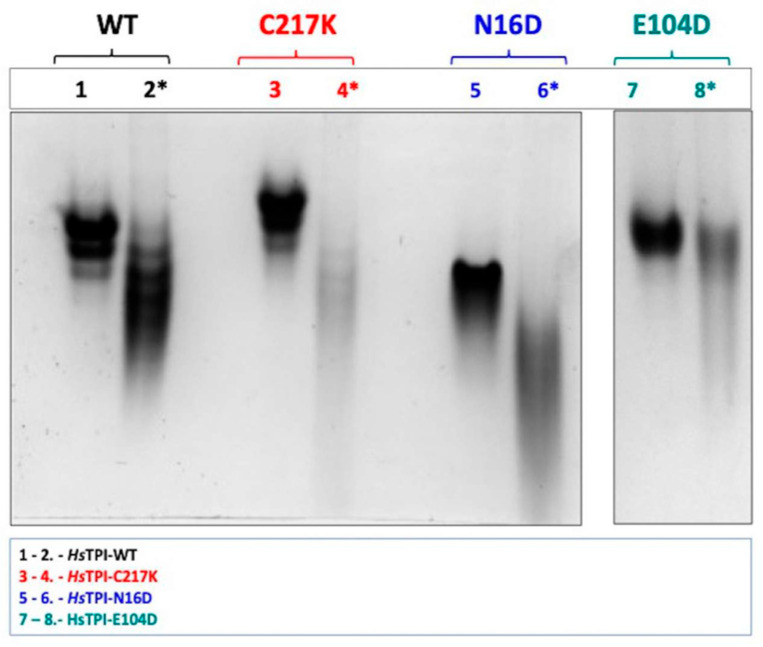
N-PAGE analysis of recombinant enzymes in the absence and presence of 1 mM G3P. Enzymes were incubated for 48 h at 37 °C. Differential electrophoretic mobility patterns were observed due to enzyme catalysis. Lanes 1 and 2 display the WT enzyme without and with G3P, respectively (black). Lanes 3 and 4 show the C217K mutant without and with G3P (red). Lanes 5 and 6 present the N16D mutant without and with G3P (blue). Lanes 7 and 8 illustrate the E104D mutant without and with 1 mM G3P (green). Each lane was loaded with 10 µg of protein, with lanes containing G3P indicated by (*). The NPAGE results are representative of three independent experiments.

We evaluated the impact of the modifications by analyzing native gels, which reveal differences in the proteins’ charge-to-mass ratios and conformational state through Native-PAGE (N-PAGE). As shown in Figure 2, the electrophoretic profiles in the presence and absence of the substrate G3P were compared. G3P binding elicited notable shifts in electrophoretic mobility in all enzymes, strongly suggesting changes in native conformation and net charge. Significant differences were observed between the HsTPI mutants; for example, in the WT enzyme, lane 2 exhibited a marked shift in N-PAGE mobility upon G3P binding. This shift was even more pronounced in the N16D mutant, as shown in lane 6. Similarly, the E104D mutant showed substantial shifts in electrophoretic mobility, particularly in lane 8 (Figure 2), correlating the effect of substrate interaction on protein structural dynamics.

Remarkably, C217K exhibited the most significant alteration in electrophoretic migration in the presence of the G3P substrate. These observations indicate a progressive and substantial structural change by molecular wear induced by different mutations in the presence of the substrate, with C217K, which has the highest enzymatic activity, producing the greatest electrophoretic distortion. Therefore, each enzyme variant has a unique influence on the native structure in the presence of G3P. The observed variations in electrophoretic mobility suggest that different mutations lead to gradual structural alterations in response to substrate binding and catalysis.

### 2.4. G3P Incubation Reduces Cys Derivatization with DTNB in Mutant HsTPIs

**Figure 3:**
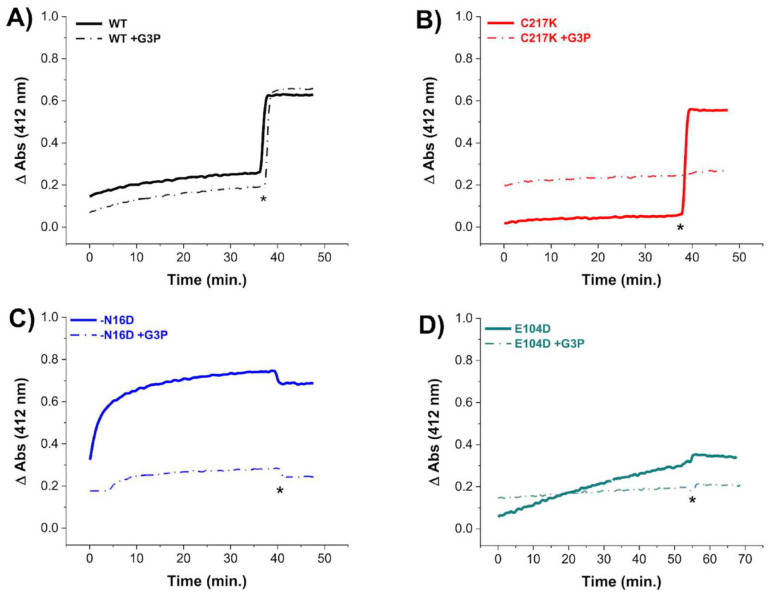
Cysteine quantification under native conditions of HsTPI WT and mutants incubated with G3P. Differential derivatization of Cys residues in HsTPI-WT, C217K, N16D, and E104D was observed in the presence of G3P. Enzymes were incubated at 200 µg for 56 h at 37 °C: (**A**) WT (black line), (**B**) C217K (red), (**C**) N16D (blue), and (**D**) E104D (green). Enzymes incubated with 1 mM G3P are represented by dashed lines, while controls are indicated by solid lines. After incubation, Cys residue accessibility was monitored with 4 mM DTNB, with readings taken at 412 nm from 0 to 60 min. Samples were denatured via the addition of SDS (indicated by asterisks). The absorbance of the blank buffer was subtracted from each sample. Experiments were performed in triplicate and yielded qualitatively identical results.

To evaluate the permeability of Cys residues in HsTPI enzymes to DTNB under exhaustive catalysis, we performed Cys titration experiments in the presence of the G3P substrate. Our results revealed decreased derivatization of Cys residues in the enzymes after prolonged incubation with 1 mM G3P (Figure 3). Notably, the C217K, N16D, and E104D enzymes exhibited resistance to Cys derivatization by DTNB (Figure 3B, Figure 3C and Figure 3D, respectively), suggesting lower accessibility to Cys modification by DTNB under catalytic conditions. For instance, in the TPI-C217K mutant exposed to the substrate, no increase in TNB formation (which results from Cys derivatization) was detected, even after the addition of a denaturing agent. This result suggests that the thiol-reactive agent was unable to access free Cys residues, potentially due to the formation of MGO adducts (arising from the degradation of G3P) with these aminoacyl residues.

In contrast, TPI-WT exhibited a significant increase in the TNB signal after the addition of a denaturing agent, suggesting that MGO formation, which could form adducts with Cys, did not occur. In contrast, for the N16D and E104D enzymes in the presence of G3P, the absence of increased TNB signal under denaturing conditions strongly suggests that Cys residues were chemically modified by MGO during exhaustive catalysis.

Based on these results, it is likely that adducts with Cys residues are generated, promoted by the catalysis of the G3P substrate in the mutant enzymes, thus preventing their derivatization by DTNB. Therefore, the structural alterations observed in these mutants facilitate the reaction of MGO with nucleophilic amino acids such as Cys.

### 2.5. CD Spectroscopy Reveals G3P-Induced Secondary Structure Alterations and Reduced Thermal Stability in N16D and E104D Mutants

**Figure 4:**
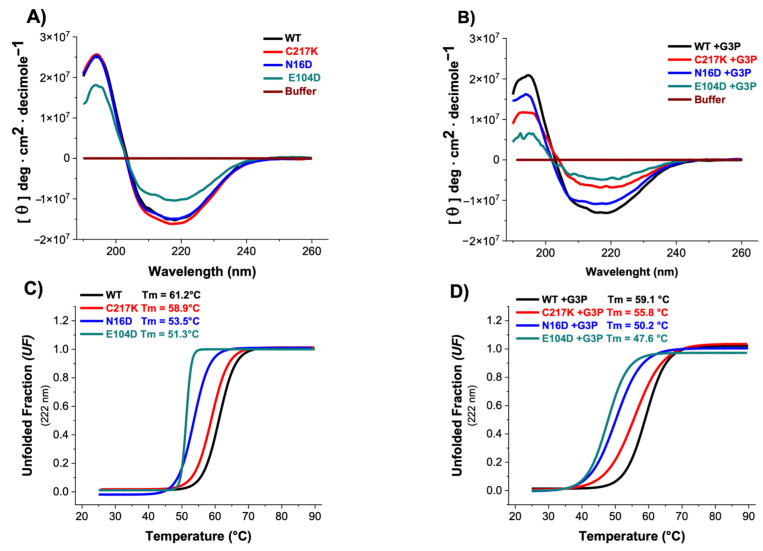
Secondary structure and global stability of HsTPI-WT and mutants. (**A**) Circular dichroism (CD) in the far-UV region for HsTPI-WT and mutants in the absence and (**B**) presence of G3P. Filled lines represent enzymes incubated with G3P, which were incubated for 24 h at 37 °C. (**C**) Simultaneously, these samples were assessed for thermal stability by measuring the change in CD signal at 222 nm as the temperature increased from 25 to 90 °C in the absence and (**D**) presence of 1 mM G3P. The fraction of unfolded protein and mean denaturation temperature (Tm) values are shown for each plot. Spectra were averaged from three replicated scans, with blanks without protein being subtracted. These experiments are representative of three independent experiments, with SE (±) < 5%.

Circular dichroism (CD) spectroscopy provides information on protein conformation. This technique measures protein secondary and tertiary structure changes caused by molecular interaction, perturbation, or aggregation, providing insights into conformational changes and protein stability. The experiments were conducted both in the absence and presence of the G3P substrate to evaluate its impact on enzyme secondary structure and thermal stability under conditions of enzymatic catalysis. As shown in Figure 4A, most enzymes exhibited similar CD profiles, except for TPI-E104D, which displayed a notable reduction in CD intensity. The presence of G3P in both E104D and N16D variants induced significant decreases in their CD spectra compared to other enzymes, as shown in Table 2, suggesting alterations in their secondary structure due to catalytic activity (Figure 4B).

Thermal stability assessments revealed that the N16D and E104D enzymes exhibited lower stability, with respective decreases of 8 and 10 °C compared to the HsTPI-WT (Figure 4C). Incubation with G3P exacerbated the thermal stability differences, particularly in E104D and N16D mutants, accentuating their alterations in global stability (Figure 4D). These results suggest that the presence of the G3P substrate induces alterations in the secondary structure and affects the thermal stability of the enzymes. Specifically, the E104D and N16D mutants appear more susceptible to modifications in their secondary structure and thermal stability, indicating a potential relationship between catalytic activity and structural integrity.

### 2.6. Fluorescence Spectroscopy Reveals the Differential Effects of Mutations on Protein Structure and ANSA Binding

**Figure 5:**
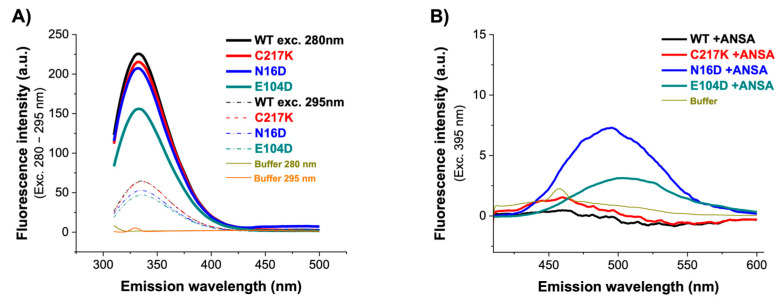
Fluorescence emission spectra of HsTPI-WT and mutants. (**A**) Intrinsic fluorescence emission spectra of WT, C217K, N16D, and E104D were recorded from 310 to 500 nm after excitation at 280–295 nm. (**B**) Extrinsic fluorescence spectra without and with 150 µM ANSA were recorded from 400 to 600 nm after excitation at 395 nm. Blanks without protein were subtracted from the experimental spectra, and each spectrum represents the average of three replicated scans.

Fluorescence emission spectra are a robust means to elucidate the relationship between protein dynamics and stability. Intrinsic fluorescence in proteins is produced primarily by exciting the protein at 280 nm, and an emission pattern is observed at approximately 350 nm. However, the emission wavelength of maximum fluorescence intensity (IFmax) can vary depending on the polarity of the environment containing mostly tryptophan. Intrinsic fluorescence defines the changes observed by modifying the signal emission of fluorophores due to increased solvent accessibility due to structural alterations. Therefore, the intrinsic fluorescence spectra of the enzymes were analyzed. As shown in Figure 5A, a marginal decrease in signal was observed for WT, C217K, and N16D. However, the most notable difference was found in the E104D enzyme, which showed a significant quenching of fluorescence at 280 nm, with a decrease of 70 arbitrary units (au) in the IFmax.

To determine alterations in global three-dimensional structure (3D), extrinsic fluorescence was evaluated using 8-Anilinonaphthalene-1-sulfonic acid (ANSA). It has a role as a fluorescent probe. ANS’s fluorescent properties will change as it binds to hydrophobic regions on the protein surface. Comparison of the fluorescence in the presence and absence of ligands can thus give information about how the binding of the ligand changes the surface of the protein. In the absence of ANSA, comparable fluorescence spectra were observed for the WT, C217K, and E104D enzymes, while an increase was evident for the N16D mutant. This indicates no discernible exposure of hydrophobic patches on the enzyme surface. However, upon the addition of 150 µM ANSA, a substantial increase in fluorescence was observed in the E104D and N16D mutants, as shown in Figure 5B and Table 3. These results suggest that the mutations have varying effects on protein dynamics and stability. The E104D seemed to be the most affected, potentially exhibiting a less stable and more flexible structure. The results suggest that the E104D and N16D mutations might lead to increased exposure of hydrophobic regions on the protein surface.

### 2.7. Temporal Analysis of Structural Alterations and Kinetics of ARGp Adduct Formation

A temporal analysis of structural alterations was performed to understand the inter-action dynamics between the enzymes and MGO. The identification of the fluorescent ar-gpyrimidine (ARGp) adduct was facilitated by a standard curve, which allowed us to determine the formation of this adduct and to estimate the mean interaction time of the protein with the MGO (Supplementary Figure S1A). Under stoichiometric conditions (1:1), a mean saturation of 50% was observed after 87 h of sample incubation at 37 °C (Supplementary Figure S1B). This was exemplified by the C217K in the presence of G3P (Supplementary Figure S2A,B) analyzed via the ARGp signal and extrinsic fluorescence assessed via ANSA. Consequently, experiments were extended up to 96 h of incubation, during which time structural alterations were identified in enzymes incubated in the presence of either G3P 2 mM or MGO 1 mM.

#### 2.7.1. Fluorescence Analysis Revealed Increased ARGp Formation and Hydrophobic Patch Exposure in Mutant TPIs upon Incubation of G3P

**Figure 6:**
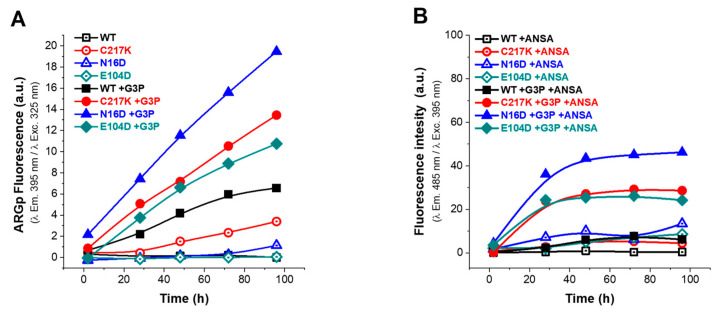
Analysis of structural effects exerted by G3P on TPI variants. Enzymes were exposed to G3P for 2, 28, 48, 72, and 96 h at 37 °C. (**A**) Protein samples of enzymes are represented as follows: WT (black squares); mutants C217K (red circles), N16D (blue triangles), and E104D (green diamonds). Each was incubated with 2 mM G3P for the specified times and then analyzed by excitation at 325 nm to obtain emission scans from 340 to 600 nm. Empty squares represent (incubation controls) (**A**) treatments without G3P and (**B**) without ANSA, while filled squares in both graphs represent enzymes incubated with G3P or ANSA. The fluorescence intensity signal is plotted at 395 nm. (**B**) The ANSA assay was performed under the same conditions, with samples recorded at emission wavelengths from 400 to 600 nm following excitation at 395 nm. The fluorescence signal is plotted at 485 nm. All assays were carried out in triplicate.

To investigate the effects of G3P catalysis on conformational stability, fluorescence assays were performed by determining the ARGp adduct signal and ANSA probe signal over 96 h of incubation at 37 °C (Figure 6).

In the absence of G3P, the fluorescence signal was minimal (Figure 6A), whereas, upon G3P addition, a progressive increase in fluorescence signal was observed, attributed to ARGp formation. As seen in Figure 6A, the ARGp formation at 96 h shows the differential effect of incubation with the substrate on the mutants vs. the WT; the N16D enzyme being the most susceptible to ARGp formation, incubation time increased its signal at 395 nm by 3-fold. C217K increased by 2-fold and E104D by 0.6-fold compared with the signal recorded for the WT in the presence of G3P. Remarkably, this increase was most prominent in N16D, followed by C217K, E104D, and WT, strongly suggesting the formation of ARGp in the mutant enzymes compared with the WT in the presence of the G3P substrate.

Regarding the ANSA signal, baseline levels were observed in the absence of G3P, with slightly higher signals in the E104D and N16D enzymes (Figure 6B). However, upon incubation with G3P, a significant increase in extrinsic fluorescence signal was noted in the mutants and was particularly pronounced in N16D, followed by C217K and E104D; meanwhile, the WT maintained a baseline signal (Figure 6B). These observations suggest a correlation between the formation of MGO adducts and exposure of hydrophobic patches in mutant enzymes, which is consistent with the substantial increase in fluorescence intensity in the presence of G3P. Mutant enzymes, particularly N16D and C217K, exhibited more pronounced changes in fluorescence signals in comparison with the WT, in terms of both ARGp formation and ANSA signal (Figure 6A,B and Figure S3(1A–3A),(1B–3B)).

#### 2.7.2. MGO Exposure Induces Enhanced ARGp Formation and Hydrophobic Patch Exposure in N16D and C217K Mutants Compared with WT TPI

**Figure 7:**
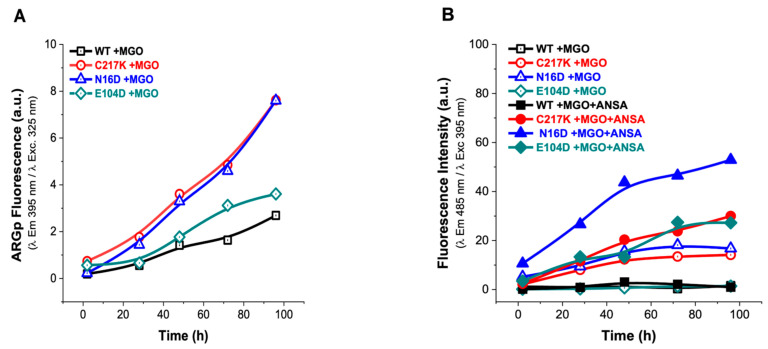
Analysis of structural effects exerted by MGO on TPIs. (**A**) Increased signal from the ARGp adduct relative to incubation time in enzymes exposed to 1 mM MGO, with readings taken at 2, 28, 48, 72, and 96 h at 37 °C. (**B**) The addition of 150 µM ANSA induced an increase in hydrophobic patches on the exposed surface of the protein over time. The empty symbols in (**B**) represent treatments without ANSA (incubation controls), while filled squares represent enzymes incubated with MGO.

Incubation assays were conducted in the presence of MGO to determine its permeability to enzymes. This analysis, as well as the previous experiment with G3P, corroborated that MGO induced differential structural alterations in each enzyme, revealing a time-dependent increase in the fluorescence signal in all TPI enzymes, peaking at 96 h of incubation with MGO (Figure 7A); this observation is consistent with the mean saturation time of 87 h for ARGp formation (Figure S3B) and exemplified by C217K in the presence of MGO (Figure S2B). Interestingly, ARGp formation was more pronounced in N16D and C217K mutants compared to the WT enzyme (Figure 7A). Additionally, the ANSA assay exhibited a significant increase in hydrophobic patches in the N16D enzyme, followed by C217K and E104D mutants. At the same time, there was no notable increase in hydrophobic patch exposure in the WT enzyme (Figure 7B). Therefore, exposure of TPI to MGO induces structural alterations, with mutants N16D and C217K exhibiting increased ARGp formation and exposure to hydrophobic patches compared with the WT.

Significantly, increased ARGp formation and increased exposure to hydrophobic patches were identified in both N16D and C217K mutants compared with the WT enzyme. These TPI variants showed similarity to the hydrophobic patches in terms of their behavior, with a 30-fold increase in the fluorescent signal for C217K, a 53-fold increase for N16D, and a 27-fold increase for E104D at 96 h. (Figure 7B). These results therefore suggest that MGO-induced modifications are more prominent in TPI mutants, highlighting the importance of understanding the impact of MGO on protein structure and function (Supplementary Figure S4(1A–3A),(1B–3B)), particularly in pathological states associated with MGO accumulation.

### 2.8. TPIs Altered at the Functional and Structural Level by G3P and MGO Are Partially Reversed by MGO Scavengers

**Figure 8:**
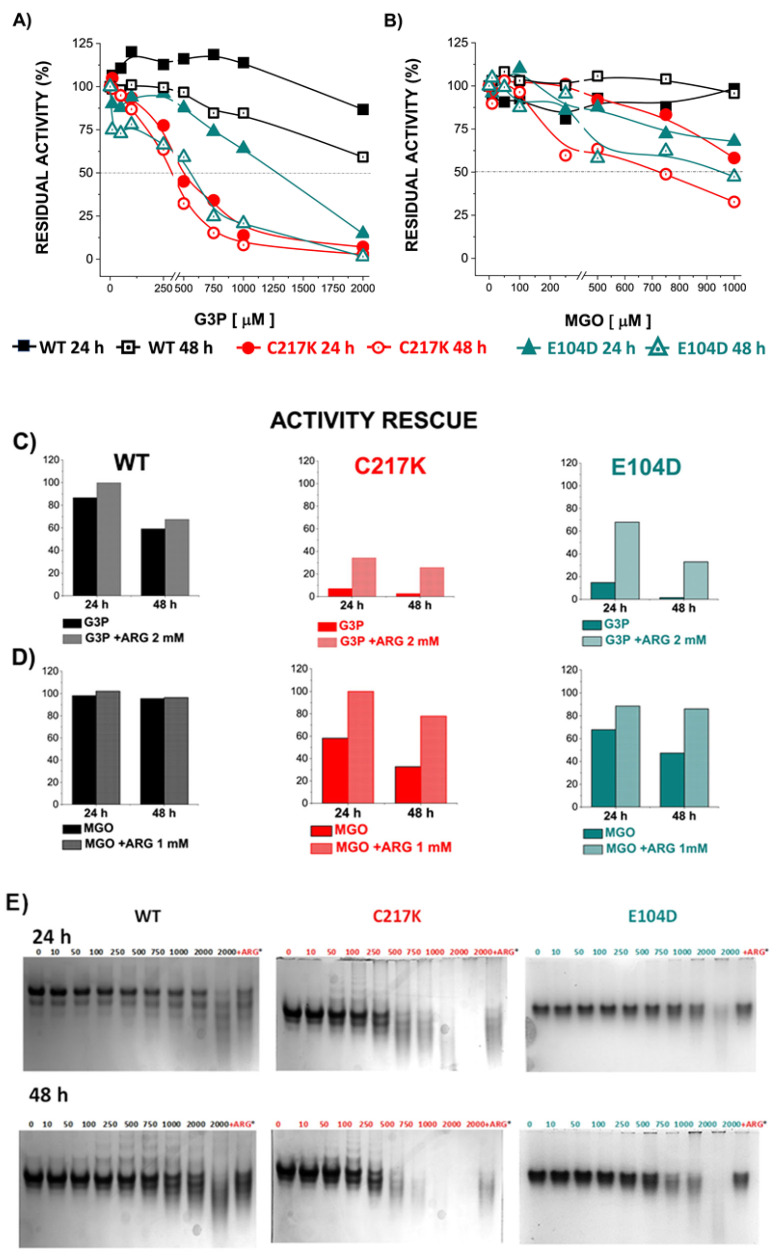

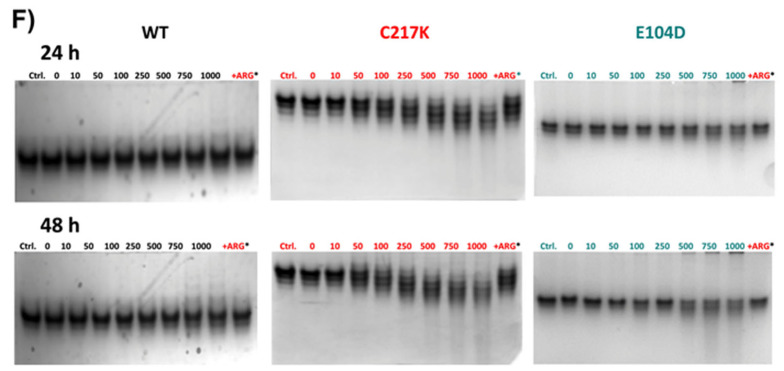
Differential kinetic and structural effects induced by G3P or MGO in HsTPI-WT, C217K, and E104D. Enzymes were incubated for 24 or 48 h with increasing concentrations of G3P (0, 10–2000 µM) or MGO (0, 10–1000 µM). (**A**) Residual activity plots for the three proteins incubated with G3P at both times: WT at 24 h (black squares) and 48 h (open black squares), C217K at 24 h (red circles) and 48 h (open red circles), and E104D at 24 h (green triangles) and 48 h (open green triangles). (**B**) Comparative residual activity of proteins incubated with MGO. (**C**,**D**) The residual activity with arginine (squared bars) for each enzyme at 24 or 48 h is compared with that of the protein without arginine at the same time (plain color bars), (**C**) Bar graphs showing the protective effect of arginine at the highest concentration of G3P (2000 µM) or **D**) MGO (1000 µM) at 24 or 48 h. (**E**,**F**) Effects of protein destabilization observed in N-PAGE induced by G3P (**E**) or MGO (**F**) over the incubation period. The differential effect of equimolar addition of arginine (asterisk) as an MGO scavenger on TPI-G3P reactions (2000 µM) or TPI-MGO (**D**) (1000 µM) for the WT, C217K, and E104D enzymes is shown in the N-PAGE. Each experiment was performed in duplicate.

Under constant catalysis, TPI enzymes can alter their structure, and MGO formation has the potential to synergistically induce further structural alteration. Therefore, experiments were conducted to determine changes in enzyme activity and electrophoretic mobility in the presence of G3P and MGO. Residual activity was determined after 24 or 48 h of exposure to these metabolites. Residual activity gradually decreased after incubation with G3P for 24 or 48 h. However, more pronounced effects were observed in TPI mutants (Figure 8A). Thus, the dynamic nature of TPI enzymes, being influenced by continuous catalysis, leads to structural alterations that can be enhanced by the formation of MGO.

However, direct incubation with MGO had a less pronounced impact on TPI activity, reaching 50% inactivation in the mutants (Figure 8B). Interestingly, the addition of the Arg scavenger demonstrated a protective effect on enzyme activity in the presence of G3P or MGO. These results underline the dynamic interplay between enzyme structure, substrate interaction, and possible protective mechanisms, which acts to maintain enzyme function under physiological stressors such as MGO.

The echimolar addition of Arg demonstrated a protective effect on enzymatic activity; hence, TPI enzymes were exposed to Arg scavenger in the presence of G3P and MGO (Figure 8C,D). We observed a protective effect on the residual enzyme activity in the mutants evaluated. Finally, the N-PAGE showed that the integrity of the electrophoretic mobility of the TPIs was gradually lost with incrementally increasing exposure to G3P and MGO (Figure 8E,F). The above results suggest that TPI enzymes undergo structural alterations under continuous catalysis, which can be further exacerbated by the formation of MGO. However, some degree of restitution in integrity was reached following exposure to Arg.

### 2.9. Structural Alterations Prompted by Formation of AGEs in HsTPI Suggests Aggregation by Protein Cross-Linking

An important consequence of MGO-mediated glycation is the generation of AGEs, including MGO-Lys cross-linking dimers (MOLDs) and arginine–lysine dimers (MODICs), among others [31]. The signal spectral data obtained by equimolar ARG-MGO interaction were monitored over 421 h (Supplementary Figure S1A). The kinetics of the ARGp50% signal reached a peak at 87 h, providing evidence of an increase in the fluorescent ARGp adduct signal (Supplementary Figure S1B). For the C217K mutant that was previously incubated with G3P or MGO, an increase in the fluorescence of the ARGp adduct was observed. These fluorometric scans were followed for 500 h (Supplementary Figure S2A,B). The aforementioned increase in fluorescence strongly suggests the formation of AGEs, which leads to protein cross-linking and subsequent aggregation.

**Figure 9:**
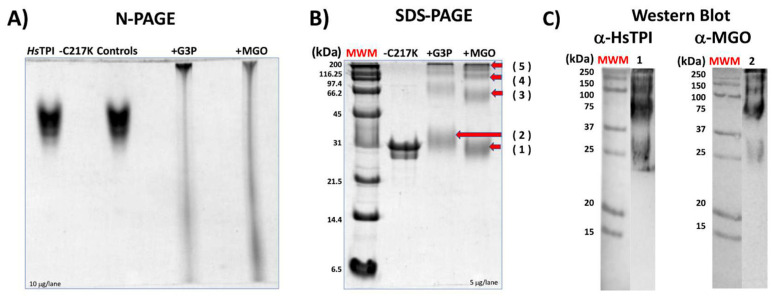
Effect of glycation by G3P or MGO on HsTPI-C217K. (**A**) N-PAGE analysis of HsTPI-C217K enzymes incubated with G3P or MGO for 500 h at 37 °C. Samples were loaded with 5 µg per lane. Controls correspond to protein incubated without G3P or MGO. (**B**) SDS-PAGE (12%) analysis. Lane 1: Relative mass standards (MWM); 5 µg of HsTPI-C217K treated with G3P or MGO was loaded per lane. (**C**) Western blot analysis of C217K treated with MGO. Blots were probed with anti-HsTPI (α-HsTPI) and anti-MGO (α-MGO) antibodies. Red arrows (1–5) indicate the molecular masses of soluble aggregates formed after exposure to G3P or MGO. Each experiment was performed in duplicate.

Prolonged incubation of TPI-C217K with G3P or MGO resulted in the formation of aggregates, which exhibited limited mobility on N-PAGE (Figure 9A). The enzymes that formed adducts with G3P or MGO showed relatively high molecular masses and were not filtered on N-PAGE (Figure 9A). This result strongly suggests the formation of gly-cation-modified species, including monomers and oligomeric forms of higher molecular mass in C217K. Notably, these soluble aggregates exhibited higher molecular masses than the tetrameric form of TPI, reinforcing the idea of glycation-mediated cross-linking with G3P and MGO (Figure 9B). Finally, Western blotting revealed these aggregates using an-ti-HsTPI and anti-MGO antibodies (Figure 9C). Thus, the formation of high-molecular-weight species resulting from glycation-induced cross-linking highlights the importance of understanding AGE-mediated protein aggregation pathways [30].

### 2.10. Structural Analysis of Ligands Accessibility in Interfacial Cavity of WT, E104D, and N16D TPIs

**Figure 10:**
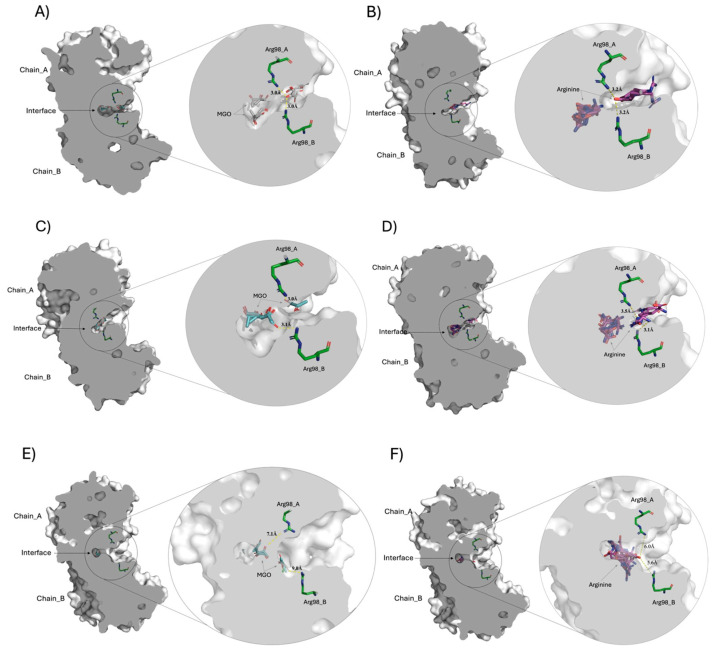
Molecular docking comparison of the crystallographic structures of TPI with different conformers of the MGO and Arg ligands. The centrally sliced molecular surface of HsTPI dimer is observed, highlighting the interface cavity between the subunits (chains A and B). Panels (**A**,**B**) correspond to docking of HsTPI-WT with MGO and Arg, respectively, revealing the ligand interactions within the interfacial cavity. Panels (**C**,**D**) display the docking results for HsTPI-E104D with the same ligands, MGO and Arg, in the interface region. Panels (**E**,**F**) correspond to the docking of the HsTPI-N16D variant with MGO and Arg, respectively. Across all models, Arg98 residues from both subunits are positioned closest to the interfacial cavity, interacting at distances between 3.0 to 9 Å. In all figures, the MGO and Arg ligands are shown in cyan and magenta colors, respectively. While the aminoacyl residue of Arg98 in both chains is shown in green color. Modeled with PyMOL version 2.5.0 (Schrödinger Inc., New York, NY, USA).

Given the observed differences in ligand accessibility among the TPIs, molecular docking studies were conducted to elucidate potential binding sites for MGO and arginine (Arg) ligands in the WT, E104D, and N16D crystallographic structures. Figure 10 illustrates the docking of these ligands, highlighting important structural features and interactions within the interfacial cavity of each enzyme.

Figure 10A,C,E shows the docking of the MGO ligand at the interfacial region of WT, E104D, and N16D TPIs, respectively. The zoomed-in images to the right of each panel provide detailed views of the interfacial cavity, where Arg98 residues from both subunits converge and make contact with MGO. Notably, the cavity volumes vary across the variants, with WT TPI exhibiting a cavity size of 105.6 Å^3^, E104D showing a similar cavity of 101.5 Å^3^, and N16D displaying a significantly larger cavity of 287.4 Å^3^ (approximately 2.7 times that of WT). These differences strongly suggest that the interfacial cavity is a critical site for the entry of low molecular weight ligands, and that the N16D variant offers the greatest accessibility for small molecules like MGO.

Figure 10B,D,F show the docking of Arg ligand in WT, E104D, and N16D TPIs, respectively. As with MGO, the right-hand zooms show the Arg98 residues interacting with the docked Arg ligand within the interfacial cavity. Interestingly, molecular docking revealed that the computational binding energies for such ligands were higher than those for MGO across all TPIs (Supplementary Table S2), underlining the strong affinity of this region for ligands with Arg-like characteristics.

The structural analysis supports the hypothesis that the interfacial cavity plays a pivotal role in ligand entry. In WT TPI, the relatively narrow cavity entrance likely impedes ligand access, whereas the wider entrance observed in E104D and N16D variants facilitates greater permeability. This structural trend correlates with experimental data shown in Figure 1, where rapid derivatization of Cys residues by DTNB was observed in E104D and N16D, compared to the slower Cys modification seen in WT and C217K TPIs. These findings further align with fluorescence studies, which indicated the formation of ARGp adducts in TPIs exposed to G3P and MGO, especially in the E104D and N16D variants.

Overall, the molecular docking results emphasize the highest permeability of the E104D and N16D enzymes for small ligands like MGO, with N16D exhibiting the highest accessibility due to its enlarged cavity. This altered interfacial region could serve as the primary site for ligand interaction, reinforcing its significance in the structural and functional dynamics of TPI.

The molecular docking analysis highlights how structural modifications in the interfacial cavity of TPI mutants modulate ligand access, selectivity, and binding affinity. The enlarged cavity in N16D correlates with a loss of enzymatic function and increased interaction with MGO, suggesting that this mutant could play a role in promoting toxic metabolite accumulation. In contrast, E104D exhibits moderate catalytic efficiency and ligand accessibility, hinting at a more subtle structural alteration with pathological potential. The molecular docking analysis thus provides a framework for understanding the structural-functional relationship of TPI mutants, with implications for developing selective stabilizers for conditions such as TPI deficiency.

## 3. Discussion

TPI is a glycolytic enzyme required for glucose catabolism, which, due to the aldol partition of hexoses, produces DHAP and G3P. It has been considered a relevant enzyme for obtaining a net yield of ATP in this pathway. Deficiency of this enzyme in humans can causes hemolytic disease (congenital non-spherocytic anemia), which is characterized by a shortage of red blood cells (anemia), movement problems, increased susceptibility to infections, and muscle weakness that can affect breathing, heart rate, and other functions. It is also responsible for the non-negligible production of methylglyoxal, considered a highly reactive cytotoxic byproduct that can alter proteins, DNA, and lipids [32].

Glycolytic enzymes, including TPI, are recognized targets of NE/PTMs, particularly through the formation of MGO adducts. Such MGO adducts arise from processes such as the carbamylation of the Arg residue at position 3 and the oxidation of the Met residue at position 14, as observed in samples from epithelial cell lines and peripheral blood lymphocytes [33]. Additionally, TPI and hemoglobin have been reported to undergo glycation in murine models, marking them as indicators of diabetes [34].

This study further elucidates how mutations in HsTPI significantly impact its kinetic stability and protein structure, promoting MGO adduct formation, which can lead to protein aggregation. This becomes relevant when generating protein misfolding and instability, which are factors that contribute to the formation of toxic protein aggregates, particularly in pathology-associated HsTPI variants [35].

Our investigation focused on comparing WT HsTPI with the mutants C217K, E104D, and the inherently unstable N16D. This analysis provides insight into how specific mutations contribute to protein unfolding and aggregation in HsTPI [36], shedding light on broader implications for protein stability. These findings are consistent with reports on other proteins, such as albumin, which exhibit similar patterns of instability and aggregation due to mutations [37].

In particular, TPI glycation has been detected in the brains of transgenic mice in a murine AD model, where β-amyloid plaque accumulates [33]. This indicates that metabolic alterations may play a role in promoting enzyme instability and glycation in neurodegenerative contexts. Our study shows that metabolite-induced inhibition and protein unfolding, whether under physiological or pathological conditions, result in significant disruptions including catalytic instability, structural unfolding, and increased resistance to proteolysis. Herein, we identified a detrimental synergy in the catalytic activity of mutant enzymes, where kinetic instability precedes protein inactivation. Specifically, the reduced substrate affinity in C217K fosters the transformation of the substrate into MGO. This compensatory mechanism accelerates the isomerization of G3P, leading to MGO adduct formation. These adducts, in turn, exacerbate the structural instability of protein, promoting further unfolding and resistance to proteolytic degradation.

Alterations in the N16D HsTPI have previously been shown to substantially reduce cellular enzyme activity and increase MGO levels in breast cancer cells (MDA-MB-231) [26]. This variant behavior in highly glycolytic cancer cells is characterized by the presence of an increased number of internal cavities and a loss of intra- and interchain non-covalent contacts, rendering a relaxed structure that is sensible to inhibitory compounds [38]. This reactivity increases susceptibility to AGEs, with the formation of ARGp adducts notably prominent. When exposed to intracellular physiological levels of MGO, this increased susceptibility leads to the inhibition and destabilization of the analyzed mutant proteins within five days.

Cys217, located in α-helix 7 of HsTPI, plays a crucial role in regulating enzyme catalysis and structural stability [39]. Mutations that disrupt interactions involving Cys217, such as C217K, can trigger MGO production and facilitate NE/PTMs in HsTPI [27]. Although the C217K mutant is similar to the WT enzyme, structural stability and enzyme catalysis are only marginally affected. In contrast, a similar substitution by directed mutagenesis in *Giardia lamblia* TPI (GlTPI-C222K) drastically impairs catalysis, introducing a positive charge, resulting in a 159-fold decrease in *k*_cat_/*K*_m_ compared to GlTPI-WT [40].

The differences in the reactivity of Cys217 in HsTPI compared to its homologue Cys222 in GlTPI can be attributed to the distinct local environments surrounding these Cys residues [40]. Nonetheless, the HsTPI-C217K mutant initially retains catalytic efficiency comparable to the WT enzyme [40]. This is consistent with changes in catalytic properties observed in both GlTPI and HsTPI, where perturbations in active site regions, specifically 212–219 and 231–234, are linked to altered enzymatic activity. Notably, the HsTPI deficiency mutant V231M, located near the active site, exhibits similar structural perturbations, further highlighting the significance of these regions in maintaining enzyme function [41].

The HsTPI-E104D mutant, associated with a human TPI deficiency, retains its normal catalytic activity but exhibits lesser substrate affinity. Structural alterations in this mutant impair both dimer formation and thermostability, leading to monomerization under conditions where the WT enzyme remains in its dimeric form [42]. This instability makes the E104D mutant, along with C217K, prone to conformational alterations induced by enzymatic activity, which promotes misfolding and MGO adduct formation. While these mutants initially maintain catalytic function, they exhibit decreased activity over time, a pattern reminiscent of other pathogenic TPI variants. This observation underscores the importance of further investigations into the molecular mechanisms driving TPI deficiency [43].

MGO is known to exacerbate cellular damage by promoting the formation of AGEs, such as ARGp, which are detectable in diabetes model animals within a month of disease onset [44]. Our results show that while the WT enzyme maintains its structural integrity in the presence of MGO, the mutants exhibit increased susceptibility to MGO permeability and subsequent ARGp adduct formation. This suggests that structural vulnerabilities in the mutants play a role in exacerbating damage under glycation conditions.

In this study, we present a straightforward biochemical approach to investigate the structural modifications in HsTPI, focusing on point mutations at key regulatory sites such as Cys217, known to induce structural instability. For instance, C217K was analyzed using these methods, providing insights into the mechanisms by which the loss of critical contacts near the mutation site differentially affects both functional stabilities. The results demonstrate that this instability leads to an initial reduction in substrate affinity and triggers significant structural alterations. These alterations were confirmed by biophysical structural probes, revealing an increased propensity for denaturation.

An important consideration is the rate of change in the secondary structural components in the mutant enzyme upon substrate catalysis. In this context, the F240L mutant, associated with human TPI deficiency, showed 6-fold higher catalytic activity in purified *Escherichia coli* extracts compared with erythrocytes from patients harboring the mutation [42]. This observation aligns with our data, suggesting that enzymatic catalysis in mutant enzymes distinctly alters their stability, rendering them more susceptible to enzymatic inactivation and structural unfolding.

These results align with observations from the F240L mutant, where the loss of the enzymatic activity can be attributed to the enzyme’s reduced ability to sustain consistent catalytic cycles in patients. This reflects enzyme exhaustion, characterized by decreased activity [45]. In our experimental setup involving G3P, we observed rapid enzyme inhibition and a lack of derivatization when exposed to both DTNB and G3P, confirming this phenomenon.

However, restoration of structural integrity was observed in the mutant enzymes under specific conditions. At the tested substrate concentration, combined with an Arg scavenger and MGO was unable to access the enzyme’s core. This prevented destabilization of the active site and inhibited the formation of ARGp adducts. Consequently, MGO did not interact with the Cys and Arg residues of the tested enzymes, highlighting the potential for protective strategies against glycation in mutant forms of this enzyme.

The presence of protein pockets is associated with increased flexibility and decreased stability, facilitating the diffusion of small ligands through globular proteins, as previously documented [46] and observed in these mutant enzymes. In this regard, it has been shown that in molecular docking studies, small ligands bind more readily to the crystallographic structure of deamidated TPI compared to the WT, due to the higher number of pockets in the deamidated enzyme [38]. Furthermore, hemi-phosphorylation of HsTPI on Ser20 in one of its subunits has been shown to create a channel that transports the substrate to the active site and functions as a switch enhancing its catalysis [47]. This supports the notion that natural modifications, such as deamidation and/or phosphorylation, can increase the formation of protein cavities, leading to more relaxed structures that allow easier access to small molecules.

In this context, our molecular docking studies conducted on WT TPI and its variants provide important insights into ligand accessibility, particularly concerning MGO and Arg ligands. These studies reveal distinct structural features and interactions within the interfacial cavity of each enzyme, underscoring the importance of this region in ligand binding. Our docking results for MGO indicate that the Arg98 residues from both subunits converge in the interfacial cavity, potentially forming contacts with MGO. Notably, the volume of this interfacial cavity varies among the TPIs, with the N16D displaying a significantly larger cavity. This substantial difference in cavity volume emphasizes its role as a critical site for the entry of low molecular weight ligands, facilitating interactions that may influence the enzyme’s functional dynamics. When analyzing the docking of Arg ligands, a similar trend is observed. The Arg98 residues again interact with the docked Arg ligand within the interfacial cavity. Interestingly, the computational binding energies for Arg consistently surpass those for MGO across all TPI variants.

This finding highlights a strong affinity of the interfacial cavity for Arg-like ligands, reinforcing the significance of this region in ligand recognition and binding processes. Thus, such findings underscore the enhanced permeability of the E104D and N16D enzymes for small ligands like MGO. The altered interfacial region in these variants could serves as a primary site for ligand interaction, thereby contributing to the structural integrity and functional dynamics of TPI. Understanding these structural dynamics is crucial for developing strategies to mitigate the adverse effects of glycation in TPI and related enzymes, especially in diseases characterized by elevated MGO levels.

Moreover, the relevance of the TPI interface extends beyond human physiology. Several studies have explored strategies that target the TPI interface in parasitic organisms to promote enzyme inactivation. For instance, research has demonstrated the perturbation of the dimer interface of TPI and its subsequent effects on *Trypanosoma cruzi* [48]. Other studies have identified novel and selective inactivators of TPI with anti-trematode activity [49] and have elucidated the structural basis for the limited response of TPI from photosynthetic bacteria to oxidative and thiol-conjugating agents [50]. These findings collectively underscore the critical importance of the TPI interface and its correlation with the loss of activity when small molecules are introduced.

A relevant question concerns the mechanism leading to the accumulation of deami-dated HsTPI in breast cancer cells, as evidenced in [26]. This phenomenon is intriguing, given that in vitro studies have shown that the recombinant deamidated enzyme exhibits greater proteolytic susceptibility [22]. A plausible explanation for this accumulation could be the NE/PTMs exerted by MGO on Cys and Lys residues. The N16D mutant, characterized by reduced enzymatic activity, increased structural relaxation, and a larger volume of internal cavities, may facilitate the access of dicarbonyl adducts, thereby interfering with proteasomal degradation.

A parallel can be drawn with the glycation of glucose-6-phosphate dehydrogenase, where resistance to proteolysis has been linked to increased conformational stability conferred by adducts (as observed in the C217K mutant exposed to MGO, shown in Figure S5), promoting greater resistance to proteolytic degradation in a glyoxal-treated fibroblast culture [51], as well as the formation of adducts such as Cys-MGO hemithioacetals and stable mercapto-methylimidazole on glycated proteins [52].

In the studied mutants that initially showed enzymatic activity comparable to the WT, instability due to a loss of catalytic function could lead to delayed cellular enzymatic turnover. This delay might result in the accumulation and transformation of substrates into MGO adducts, such as AGEs. These covalent AGE modifications cause functional impairment and promote the formation of toxic protein inclusions [53], which are likely linked to new moonlighting activities, as reported in [44], and the emergence of pathological functions. This condition promotes the development of chronic degenerative diseases by fostering the accumulation of MGO adducts, which indirectly modify and significantly disrupt the intracellular redox system.

Our report aims to highlight the potential role of TPI mutations in chronic degenerative diseases. This phenomenon is partly attributed to inefficient substrate isomerization, leading to increased MGO levels. This occurs due to the inhibition of isomerase activity or possibly the acquisition of methylglyoxal synthase activity, as previously proposed for yeast TPI enzymes, where cleavage in loop 6 of the active site converts the enzyme into a more efficient methylglyoxal synthase [54].

It has been proposed that the severity of TPI deficiency due to various point mutations is best predicted by alterations affecting the enzyme’s structural conformation and folding. While this proposal has been well-supported, it does not fully consider catalysis-promoted protein aging in TPI. Our study provides evidence that distinctive signatures related to enzymatic catalysis should be considered when studying mutations associated with TPI deficiency. Specifically, our findings reveal that catalysis-induced structural changes can differentially age mutant enzymes over time.

In the context of catalytic enhancements, previous studies have identified two compounds, resveratrol and itavastatin, that increase levels of the mutant TPI-E104D protein in a human cell model of TPI deficiency and in cells from TPI-deficient patients [55]. These findings suggest that these compounds have strong potential for protein restoration. The observed increase in protein levels in TPI-deficient TPIQ181P/E105D cells indicates that these compounds might benefit not only patients with common mutations but also those with compound heterozygous mutations [56]. Resveratrol shows promise as a near-term treatment option for patients suffering from TPI deficiency. Thus, the protein rescue observed in our experiments with Arg scavengers may help prevent protein deterioration caused by small toxic molecules.

It is evident that NE/PTMs likely underlie structural alterations in HsTPI related to enzymatic catalysis. Glycation can lead to protein misfolding, unfolding, inactivation, aggregation, and decreased turnover. Proteins with reactive Cys residues, which can form thiolate anions at cellular pH, are particularly susceptible to redox imbalances induced by ROS due to nutritional changes [57]. Excessive or chronic consumption of high-calorie foods can exacerbate these post-translational modifications, leading to aggregate formation and a proteotoxic cellular environment.

A limitation of our study is the lack of specific identification of the MGO-generated adducts in HsTPI involved in the development of NE/PTM and aggregates. To address this, we plan to incorporate advanced techniques in the near future, such as mass spectrometry, to determine the presence of MGO adducts accurately and further elucidate the role these modifications play in the pathogenesis of chronic degenerative diseases.

## 4. Materials and Methods

### 4.1. Construction of C217K Mutant and Expression and Purification of WT and N16D and E104D Mutant Enzymes

The C217K mutant was constructed by site-directed PCR mutagenesis using the wt-*hstpi* gene cloned in the pET-HisTEVP plasmid as the template [24]. The mutagenic oligonucleotides used were: Forward 5′-ggcaaccaaaaaggaggagctg-3′ and Reverse 5′-cagctcctttttggttgcc3′. External T7 promoter and T7 terminator oligonucleotides were also used. Oligonucleotides were synthetized by the Unidad de Biología Molecular, Instituto de Fisiología Celular, UNAM. PCR mixtures contained 100 ng of wt-*hstpi*, 0.2 mM dNTPs (Thermo Scientific, Waltham, MA, USA, Cat. No R0181), 100 ng of mutagenic oligonucleotides, 1.5 mM MgCl_2_ and 0.02 U/µL of Phusion High-Fidelity DNA Polymerase (Thermo Scientific, Cat. No F530S). Mutagenesis was performed using the following PCR conditions: 94 °C for 4 min, 20 cycles of 1 min at 94 °C, 1 min at 55 °C, 1 min at 72 °C, followed by a final extension at 72 °C for 10 min. The PCR product was cloned into the pJET 1.2/blunt cloning vector (Thermo Scientific, Cat. No K1232). Successful mutagenesis was confirmed by automated DNA sequencing. The gene was subcloned into the pET-HisTEVP vector after digestion with *Nde*I and *Bam*HI (New England, BioLabs, Ipswich, MA, USA, Cat. No R0111S and R0136S); introducing a (His)6-tag and tobacco etch virus protease recognition sequence at the NH-terminus of the protein. For WT-HsTPI, N16D, E104D, and C217K, expression was performed in the *E. coli* BL21-CodonPlus (DE3)-RIL strain, and protein purification was carried out by immobilized metal ion affinity chromatography as previously reported [22].

### 4.2. Protein Concentration

Protein quantification within each purification step was performed using the bicinchoninic acid method [58], with bovine serum albumin (BSA) (BSA, Fraction V, EUROClone Ltd., Devon, UK; Cat. No. EMR086050) as standard. Protein concentrations of the WT enzyme and its single mutants C217K, N16D, and E104D were calculated using ε280 nm = 32,595 M^−1^∙cm^−1^, and for proteinase K (Sigma-Aldrich, St. Louis, MO, USA, Cat. No. P2308-100MG), this value was ε280 nm = 33,380 M^−1^∙cm^−1^.

### 4.3. Enzymatic Activity

We utilized recombinant HsTPI-WT and mutant C217K, N16D, and E104D enzymes of ≥95% purity. Their enzymatic activity was determined using a coupled system involving the disappearance of NADH (MERCK, Darmstadt, Germany, Cat. No. N1161) at 340 nm [24]. The readings were carried out using a Cary 50 spectrophotometer (Varian, Cary 50, Palo Alto, CA, USA) with 5, 3.3, 5, and 60 ng/mL of WT, C217K, E104D, and N16D enzymes, respectively.

#### Kinetic Constants of the Enzymes

For the WT and the C217K mutant, *V_max_* and *K*_m_ were obtained by fitting the initial velocity data (G3P concentrations from 0.02 to 4 mM) to the Michaelis–Menten equation (*Vi* = V_max_ [S]/K_m_ + [S]) for non-linear regression calculations. The data could not be fitted for the N16D mutant, as it exhibited no saturation at the highest substrate concentration, and V_max_ and K_m_ values were not calculated. For the WT and C217K, k_cat_ values were derived from *V_max_*, considering a monomer MM of 26682.43 Da. For the N16D mutant, k_cat_/K_m_ ratios were obtained from the slope of the double reciprocal plots according to k_cat_/*K*_m_ = 1/m∙[enzyme] (where m represents *K*_m_/*V_max_*) and through the use of the previously indicated MM.

### 4.4. Native (N-PAGE) and Denaturing (SDS-PAGE) Electrophoresis

It is possible to estimate the structural stability of the enzymes by incubating them at a concentration of 1 mg/mL for 48 h at 37 °C in the presence of 1 mM G3P (its substrate). The samples were subjected to N-PAGE electrophoresis, which allows recognition of the integrity of the quaternary structure of the protein and the homogeneity between the mass–charge ratio. These gels were prepared with 7% acrylamide (total monomer concentration ([%T], in g/100 mL) and 0.226% bisacrylamide (crosslinker ([%C], in g/100 mL), and the electrophoresis was performed in Tris–glycine buffer pH 8.5 (12.5 mM Tris, 96 mM glycine) according to the method described by [59] and modified by [22,24]. Protein samples containing 5 to 10 μg of protein, both incubation controls and subjected to catalysis by addition of substrate (5–10 μg), were incorporated in 40% glycerol and run in paired lanes for 3 h at constant 7 mA and subsequent staining with Coomassie brilliant blue G [22].

The sodium dodecyl sulphate–polyacrylamide gel electrophoresis SDS-PAGE (von Jagow) were performed in Tricine–SDS [60]. The gels were made in acrylamide at 12 or 16%. The protein samples were loaded and run by 2.5 h at 185 Volts. MM SDS-PAGE Standards, Broad Range, BioRad, Hercules, CA, USA, Cat. No. 161-0317 (6.5–200 kDa). The gels were then stained with colloidal Coomassie [61].

### 4.5. Titration of Free Cysteines

Initial substrate-free enzyme permeability by derivatization of free cysteines was carried out under native conditions using Ellman’s reagent; briefly, TPI recombinant enzymes (330 μg/mL) were added to the quartz cuvette in 100 mM Triethanolamine (TE) buffer (SIGMA, St. Louis, MO, USA, Cat. No. T1502-500G) at pH 7.4 and 25 °C. After addition of 4 mM of the cysteine-derivatizing reagent, 5,5′-Dithiobis(2-nitrobenzoic) acid (DTNB) (SIGMA, Cat No. D8130-5G), ε = 13,600 M^−1^∙cm^−1^ at 412 nm and pH 7.4, Cys modification was determined by recording ΔAbs at 412 nm for each enzyme [62]. Readings were recorded at 25 °C using a Cary 50 spectrophotometer, and TNB formation was recorded every 30 s. After 60 min, 20% SDS was added to denature the protein and expose non-accessible Cys residues to the derivatizing reagent. Abs were measured at 412 nm. The blank containing 4 mM DTNB was subtracted from each sample to give the net increase in Abs.

#### Susceptibility to Cysteine-Derivatizing Reagent After Substrate Incubation

Protein samples with 200 μg of protein at 1mg/mL incubated for 54 h at 37 °C in TE buffer at pH 7.4 without or with G3P 1 mM in a recirculating bath (LAUDA, Lauda-Königshofen, Germany, Brinkman Ecoline RE106). At the end of the incubations, the protein samples were derivatized. Therefore, the samples in 600 μL were exposed to 4 mM DTNB. Subsequently, readings of the ΔAbs at 412 nm due to TNB formation were recorded after 60 min. SDS 20% was added to expose the non-accessible Cys residues to the derivatizing reagent. Readings were recorded at 25 °C. The Abs at 412 nm of the blank containing 4 mM DTNB was subtracted to obtain the net increase in Abs.

### 4.6. Circular Dichroism Spectroscopy (CD)

#### 4.6.1. Far-Ultraviolet CD (Far-UV CD)

The CD spectra were analyzed in order to identify changes in the secondary structure of the enzymes. Samples at 1 mg/mL of control and plus-G3P 1 mM were incubated for 24 h at 37 °C. Protein samples were pre-dialyzed in 25 mM NaH_2_PO_4_ buffer at pH 7.4, diluted to 0.1 mg/mL, and analyzed using a 0.1 cm quartz cuvette at 25 °C. CD spectra were acquired using a JASCO J-810 spectropolarimeter (Jasco Inc., Easton, MD, USA). Spectra were recorded over a spectral range between 260 and 190 nm, with a data pitch of 0.1 nm, continuous analysis mode, a speed of 20 nm/min., a response time of 2 s, a bandwidth resolution of 1 nm, and a total of 3 samples used to obtain CD spectra. The signal of the CD spectra of the buffer was subtracted from each sample spectrum. The results were expressed as molar ellipticity (θ), which is defined as

θ = θobs (MRW) × (100)/lc

where the observed degrees (θobs) are the average ellipticity of the residues observed in degrees, (c) is the protein concentration in mg/mL, (MRW) is the average relative mass of the aminoacyl residues (106.72972 Da), and (l) is the path length expressed in cm. The CD spectra obtained were the averages of five scans. The spectra were smoothed through an internal algorithm using the Jasco software package J-810 for Windows.

The online software BeStSel Protein Circular Dichroism Spectra Analysis Online Software (v1.3.230210) was used to estimate the content of secondary structures via deconvolution of the spectra obtained for the CD of each enzyme in the far-UV range (Beta Structure Selection) [63].

#### 4.6.2. Thermal Stability

For the evaluation of protein thermal stability, protein unfolding was assessed via the change in CD signal at 222 nm according to a scan from 25 to 90 °C following incremental increases in temperature of 24 °C/h. The unfolded protein fraction and mean denaturation temperature (T_m_) values were calculated by recording molar ellipticity at 222 nm. HsTPI at 100 μg/mL was previously dialyzed for the experiments using NaH_2_PO_4_ 25 mM buffer. From the data obtained, the apparent fraction of denatured subunits (FD) was calculated using the Boltzmann equation, as follows:

FD = yN − y/yN − yD

where yN and yD are ellipticity values of the native and unfolded fractions, respectively. Both parameters were linear extrapolations of the initial and terminal portions of the curve as a function of increasing temperature.

### 4.7. Intrinsic Fluorescence Spectroscopy

Protein controls HsTPI-WT, C217K, and N16D at 1 mg/mL were previously dialyzed in 100 mM TE buffer at pH 7.4 and incubated for varying periods of time (0, 2, 28, 48, 72, and 96 h) at 37 °C without G3P or MGO. Aliquots were removed each time, and the readings were analyzed at a dilution ratio of 1:20 in buffer TE. Each fluorescence intensity reading was obtained using a Perkin-Elmer LS-50 spectrofluorometer (Waltham, MA, USA) in a quartz cuvette with a 1:20 diluted sample (protein at 0.05 mg/mL) in 700 μL. The fluorescence signal of the buffer was subtracted from the protein spectra. Measurements were based on a triplicate of three individual protein preparations. Scans were recorded from 310 to 500 nm and λ exc. at 280 and 295 nm to obtain emission spectra. Exc. and em. slits of 3.5 and a scan speed of 100 nm∙min^−1^ were utilized.

The initial analysis of intrinsic fluorescence (IF) allowed us to determine the changes in maximum fluorescence intensity expressed in arbitrary units (IFmax, a.u.) of each sample of enzyme control. The wavelength emission (λ em.) of IFmax in nm (λmax) was calculated by selecting the point that exhibited the highest FI expressed in arbitrary units (a.u.); the spectral center of mass (SCM) wavelength was calculated according to the method of [64], where (λ) is the wavelength used and (I) is the intensity value obtained at each wavelength. In the same form as controls, readings (0 and until 96 h) were performed in order to facilitate a kinetic demonstration of the structural changes induced by both compounds in HsTPI-WT, C217K, and N16D enzymes exposed to G3P or MGO. The results of the fluorescence spectra were then plotted (PC Software, OriginPro 2021b, Northampton, MA, USA).

#### 4.7.1. Analysis of the Protein Formation in the Fluorescent Adducts of ARGp

##### Control Curves of the ARGp Adduct Formation

Based on the method reported by Shipanova et al. [65], control curves of ARGp formation were produced with Arg-MGO as a reference to ARGp formation (ARG-MGO; SIGMA, Cat. A5006-100G and M0252-25ML, respectively) or Arg-G3P (Arg-G3P; SIGMA, Cat. No. G5376-1G); each sample included an equimolar concentration of 20 mM of each reactant. Sample reads were in a dilution ratio of 1:20 (G3P or MGO, 1 mM). Similarly, C217K protein samples of 1 mg/mL vs. TE buffer at pH 7.4 were incubated for 0 to 500 h at 37 °C with G3P or MGO at 20 mM.

#### 4.7.2. Alterations to the Three-Dimensional Structure of G3P or MGO Induced and Evidenced by Intrinsic Fluorescence

IF was similarly used to determine the structural effect exerted, over time, on enzymes incubated with 2 mM G3P or 1 mM MGO.

##### Kinetics of ARGp Fluorescent Adducts’ Formation in WT and Mutant Enzymes

Protein samples of HsTPI-WT, and C217K, and N16D mutants were treated with G3P or MGO (as in the six-point method described above) but were incubated with 2 mM G3P or 1 mM MGO (2, 28, 48, 72, and 96 h) at 37 °C before being analyzed by λ exc. at 325 nm to obtain λ em. scans at 340–600 nm. The IFmax ARGp λ em. at 395 nm evidenced the formation and growth in time of the fluorescent adducts of ARGp in the enzymes, and a concomitant loss of IFmax and shift in SCM over time were observed in the treated samples vs. their controls. Thus, the formation of ARGp was associated, as previously described [62], with the unfolding and alteration of the protein structure.

##### Extrinsic Fluorescence Assays

We determined extrinsic fluorescence (EF) using hydrophobic patches exposed to the protein surface, as evidenced by 8-Anilino-1-naphthalenesulfonic acid (ANSA) (SIGMA, Cat. No. A1028-100G) in the protein samples exposed to 2 mM of G3P or 1 mM of MGO (the samples were treated using the six-point method described above). On this occasion, however, we recorded λ em. 400 to 600 nm with λ exc. 395 nm; the final concentrations of ANSA and HsTPI were 150 μM and 327 μg/mL, respectively, as previously reported [24]. From the λ em., the fluorescence signal at 485 nm was used to plot the binding of ANSA to the protein over time.

### 4.8. Effect of Physiological Concentrations of MGO or G3P on HsTPI-WT Enzymes and C217K and E104D Mutants

Experiments were performed with 15 μg enzyme samples incubated at 1 mg/mL with different concentrations of MGO or G3P, from 0, 10, 50,100, 250, 500, and 750 to 1000 and 2000 μM. The purpose of such experiments was to determine the effect of adding the MGO scavenger to the protein samples at the highest concentrations of G3P or MGO (to which iso-stoichiometric Arg was added at 1000 μM or 2000 μM, respectively). Arg was used to prevent protein glycation with an MGO scavenger. Samples were incubated in TE buffer for 24 and 48 h at 37 °C in a recirculating bath (LAUDA, Brinkmann EcoLine RE106). In the end, residual TPI activity was determined. Native-PAGE (N-PAGE) was simultaneously performed to observe the charge of the quaternary structure and any structural instability due to alterations in protein.

#### 4.8.1. Enzyme Activity

The residual activity of HsTPI-WT and mutant C217K and E104D enzymes was determined after each increment in incubation time in order to observe enzymatic inhibition within the intracellular range of normal concentrations of G3P or MGO explored in the enzymes. To determine residual enzyme activities, 1 mM of G3P substrate was added to the coupled reaction.

#### 4.8.2. Enzyme Stability and Migration Patterns in the Native- and SDS-PAGE of Glycated TPIs

After incubation of HsTPI-WT, C217K, and E104D for 24 or 48 h at 37 °C, N-PAGE tests were run for 3 h at 7 mA, both in 13 mM Tris buffer (Tris, MP Biomedicals, Tokyo, Japan, Cat. No. SKU:02103133.1) and 96 mM glycine (J.T. Baker, Cat. No. 4057-02) at pH 8.5 to show changes in quaternary structure or charge, unfolding and aggregation as a result of G3P or MGO; we used 5–10 μg of protein per lane. Electrophoresis was carried out in an electrophoretic chamber (Hoeffer SE250 Mighty Small II Mini Vertical Electrophoresis Unit, San Francisco, CA, USA) with 0.75 mm thick gels to determine the effect of G3P or MGO treatments on the structural integrity of the enzymes vs. the control (without treatment).

### 4.9. Western Blotting of Enzymes Exposed to MGO and G3P

Extensive alterations of C217K, N-PAGE, and SDS-PAGE were carried out to show any structural alteration and instability due to the formation of adducts. Protein samples of C217K mutant previously incubated with G3P or MGO (20 mM) for 500 h at 37 °C were loaded at 5 μg/lane to an N-PAGE test to identify any alterations due to the formation of adducts and modifications in quaternary structure; 10 μg/lane in SDS-PAGE at 12% were used to evidence aggregates induced by incubation with G3P or MGO for 500 h in C217K enzymes.

Protein samples of the enzyme C217K incubated with MGO or G3P (20 mM) were revealed, and the presence of ARGp adducts was evidenced by Western blotting vs. anti-MGO and anti-HsTPI. Briefly, 1.5 μg of protein was loaded per lane onto SDS-PAGE 12%, and lane 1 included pre-stained relative mass standards (MWM) (BioRad Kaleidoscope™ Pre-stained Protein Cat. No. 1610375). Electrophoresis was carried out for 0.5 h to 90 V and 65 min at 185 V.

Electrophoretic SDS-PAGE gel, as well as PVDF membranes (Amersham™ Western blotting membrane Hybond^®^P, PVDF 0.22 μm, Sigma-Aldrich, Cat. No. GE10600021), were embedded in absolute methanol (J.T. Baker grade HPLC, Capitol Scientific, Austin, TX, USA, Cat. No. 9093-03) and equilibrated in transfer buffer Trizma-base 24 mM, (SIGMA, Cat. No. T1503-500G); 192 mM glycine (MP Biomedicals, Cat. No. 194825); 0.1% SDS (SIGMA Cat. No. L4390-500G) and 20% methanol at pH 8.3 for 15 min of shaking. The PVDF membrane was transferred for 1 h at 20 V. in a horizontal transfer chamber (Bio-Rad, Hercules, CA, USA, Trans-Blot^®^ SD Semi-Dry Electrophoretic Transfer Cell Cat. No. 170-3940).

After transfer, the membranes were blocked with TBS-Tween-20 buffer (TBS-T) + 8% BSA at pH 7.6, undergoing constant shaking for 1 h at room temperature. TBS-T buffer (10 mM Trizma-Base; 150 mM NaCl (VWR, Leicestershire, UK, Cat. No. 0241-2.5KG); 0.1% Tween 20) was used to perform 2 washes, each lasting for 10 min.

The membranes were incubated in TBS-T (0.1%) + BSA 1% overnight at 4 °C and shaken (10 mL) with 1st Ac anti-methylglyoxal (α-MGO) (ABCAM, Waltham, MA, USA, [9F11] ab243074) at a dilution of 1:1000 or with 1st Ab anti-HsTPI (α-HsTPI) 1:1000 (SantaCruz H11, Dallas, TX, USA, Cat. No. sc-166785 HRP). After incubation, membranes were washed three times with TBS-T at room temperature, with each wash lasting 10 min.

Subsequently, 2nd Ab, peroxidase-bound anti-mouse IgG (Cell Signaling HRP-linked, Danvers, MA, USA, Cat. No. 7076s) was added in TBS-T at 1:3000 + BSA 1% for 1 h at room temperature and shaken; subsequently, three washes with TBS-T were performed for 10 min each. Detection of target proteins was carried out with luminol Santacruz Biotec (ImmunoCruz Western Blotting Luminol Reagent, Cat.No. sc-2048; Santacruz Biotechnology, Inc., Dallas, TX, USA) in a transilluminator (Bio-Rad, ChemiDoc XRS^+^ Gel Imaging System).

### 4.10. Refractory Proteolysis in C217K Glycated by MGO

Initially, the spectra data signals obtained by interaction equimolar ARG-MGO were assessed for 421 h (Supplementary Figure S1A). These samples were recorded at λ exc. at 325 nm and λ em. from 340 to 600 nm. These results were plotted, and the IF at λ em 395 nm was plotted against time to generate a saturation curve fitted by the Michaelis–Menten equation in order to obtain the kinetics of the ARGp50% signal at 87 h; this allowed us to evidence the increase in ARGp fluorescent adduct signal. In previously glycated HsTPIC217K incubated with G3P or MGO for 500 h, we observed an increase in the ARGp adduct fluorescence.

Fluorometric scanning of enzymes was carried out for 500 h. These samples were also analyzed for limited proteolysis of AGE-modified C217K enzyme vs. WT HsTPI and C217K enzymes without exposure to MGO but incubated for 500 h with proteolyzed vs. proteinase K.

The proteolytic susceptibility of C217K without or with MGO was assessed to identify the higher MM species as aggregates that could be refractory to proteolysis; the WT and C217K samples emerged as promising candidates of proteic aggregation. Briefly, to analyze proteolysis kinetics, a mol.–mol. ratio of 1:0.2651 mg/mL was used, and WT controls, C217K, and C217K with MGO were incubated in proteinase K (Sigma-Aldrich, Cat. No. P2308-1G) for varying periods of time (0, 30, 65, 130 180, 210, 240, 270, and 300 min.) in a recirculation batch at 30 °C. The reactions of proteolytic digestions were stopped with 5 mM phenylmethylsulfonyl fluoride (PMSF, SIGMA, Cat. No. 78830-5G). After the digestion reactions were stopped, the residual activity of the controls and proteolyzed HsTPIs was determined for both enzymes, according to Section 4.3. The samples were loaded in a 16% SDS-PAGE to which 0.4 M DTT had been previously added, before being boiled for 5 min. This method has previously been described in [22].

### 4.11. Molecular Docking of WT, E104D, and N16D TPI with MGO and Arg Ligands

Molecular docking studies were conducted using the crystallographic coordinates of WT-TPI (PDB code: 2jk2), TPI-E104D (PDB code: 2vom), and TPI-N16D (PDB code: 4unk), downloaded from the Protein Data Bank (PDB) [22,42]. Solvents, ions, and ligands were removed from the structures using PyMOL version 2.5.0 (Schrödinger, LLC, New York, NY, USA). The protein structures were then energetically minimized using UCSF Chimera software, version 1.18 [66], and the new minimized coordinates were used for subsequent docking calculations.

The ligands utilized in this study were MGO and arginine (Arg), both of which were selected for their biological relevance to the function and structural alterations observed in TPI variants. Their molecular structures were obtained from the ZINC database [67] (https://zinc.docking.org) and energy-minimized using Avogadro version 1.2. Hydrogen atoms were added to the protein structures, and Kollman charges (6.00 and 3.999, respectively) were assigned using AutoDock Tools (ADT) version 1.5.6 [68].

Molecular docking was performed using the “CB-Dock” server [69], which was configured with default parameters to identify potential binding sites within the interfacial cavity of each TPI variant. The resulting docking output files were saved in .pdb format for further analysis. Interactions between MGO or Arg and the interface regions of the TPI variants (WT, E104D, and N16D) were analyzed and visualized using the PyMOL Molecular Graphics System (version 2.5.0, Schrödinger, LLC, New York, NY, USA).

## 5. Conclusions

Our study demonstrates that diverse HsTPI mutations lead to enzymes’ kinetic and structural instability. These findings align with the existing literature, which indicates that similar enzymes are associated with TPI deficiency in patients or are present in highly glycolytic cancer cells with low turnover and accumulation.

The analyzed mutant TPIs exhibited reduced structural stability and shared several traits, including increased molecular permeability and diminished substrate affinity. These changes contribute to the substrate’s accumulation and subsequent transformation into MGO. Our results suggest that the exposure of the substrate G3P or the toxic metabolite MGO triggered structural and functional changes in HsTPI mutants, making them more susceptible to MGO and increasing ARGp adducts, thereby leading to lower structural stability. Furthermore, inhibition of TPI and its formation of adducts with nucleophilic residues lead to the generation of NE/PTMs. These modifications can be monitored over time through the formation of ARGp and can be detected using fluorescence techniques.

We suggesting exploring the use of small-molecule supplements that can enzymati-cally rescue and correct protein misfolding. These supplements may function as glycation scavengers to neutralize MGO and act as pharmacological chaperones, potentially reversing TPI deficiency in humans and aiding in the prevention and treatment of chronic and degenerative diseases.

## Figures and Tables

**Table 1:** Kinetic parameters in WT-HsTPI, C217K, N16D, and E104D mutants.

Enzyme	*V_max_* (μmol·min^−1^·mg^−1^)	*K*_m_ G3P (mM)	*k*_cat_ (10^5^ M·min^−1^)	*k*_cat_/*K*_m_ (108 M^−1^·s^−1^)
HsTPI-WT ^(^*^1)^	4091 ± 88	0.4	2.2	2.8
C217K	13,432 ± 1046	1.7 ± 0.3	7.1	2.4
N16D ^(^*^2)^	4229 ± 210	ND	ND	0.2
E104D	5905	0.9	3.1	2.1

*k*_cat_ and *k*_cat_/*K*_m_ were derived from *V_max_*, and *K*_m_ values were calculated from the non-linear fit of initial velocity data to the Michaelis–Menten equation. The WT and the C217K mutant data were processed similarly. * Data reported by *^1^ [24] and *^2^ [22].

**Table 2:** Circular Dichroism Spectra Deconvolution Data.

Enzyme	HsTPI-WT		HsTPI-C217K		HsTPI-N16D		HsTPI-E104D	
	Control	+G3P	Control	+G3P	Control	+G3P	Control	+G3P
Wavelength	200–250 nm		200–250 nm		200–250 nm		200–250 nm	
(Scala-factor)	1	1	1	1	1	1	1	1
Helix 1 (regular)	55.4	58.4	71.4	18.4	59.1	51.7	54.3	10
Helix 2 (distorted)	22.8	12.6	11	14.4	21.5	22.1	19.3	6.8
Anti 1 (left-twisted)	0	0	0	0	0	0	0	0
Anti 2 (relaxed)	0	0	0	0	0	0	0	0
Anti 3 (right-twisted)	14.7	15.6	12.1	0	16.2	15.9	15.2	3.6
Parallels	6.7	13.3	5.5	17.2	0	1.4	9.2	18.8
Turns	0.4	0	0	7	3.1	3.7	2.1	8.9
Others	0	0	0	42.9	0	6.2	0	51.9
Helix	78.2	71.1	82.4	32.8	80.6	72.8	73.5	16.8
Antiparallels	14.7	15.6	12.1	0	16.2	15.9	15.2	3.6
Parallels	6.7	13.3	5.5	17.2	0	1.4	9.2	8.9
Turns	0.4	0	0	7	3.1	3.7	2.1	8.9
Others	0	0	0	42.9	0	6.2	0	51.9

The percentage changes of the secondary structure components of HsTPI-WT, C217K, N16D and E104D are shown. The spectra were processed with BeStSel Protein Circular Dichroism Spectra Analysis Online Software (v1.3.230210). The table shows the percentage of the secondary structure obtained for the controls of WT, C217K, and N16D enzymes and +G3P 1 mM for 24 h at 37 °C.

**Table 3:** Intrinsic fluorescence parameters of HsTPI-WT, C217K, N16D, and E104D mutants.

Intrinsic Fluorescence Wavelength (λ exc.)		HsTPI			
		WT	C217K	N16D	E104D
280 nm	IFmax (a.u.)	225.6	215.3	207.3	155.9
	λmax (nm)	332.5	332.5	332	333
	SCM (nm)	346.9	347.1	350	347.45
295 nm	IFmax (a.u.)	64.7	63.8	52.9	47.14
	λmax (nm)	336	335	335.5	336.5
	SCM (nm)	350.5	350.7	356	350.5

## Data Availability

Data are contained within the article and Supplementary Materials.

## Supplementary Materials

The following supporting information can be downloaded at: https://www.mdpi.com/article/10.3390/molecules29215047/s1. Table S1. Quantification table of free Cys derivatized by the monomer. Figure S1. Standard curve of fluorescent ARGp adduct formation. Figure S2. Kinetics of ARGp adduct formation in HsTPI-C217K. Figure S3. Analysis of structural effects exerted by G3P on TPIs. Figure S4. Analysis of structural effects exerted by MGO on TPIs. Figure S5. Refractory proteolysis in C217K glycated by MGO. Table S2. Predicted computational binding energies of MGO and Arg ligands to interfacial pocket in TPIs.
