# Supplementary material for: Methylglyoxal-Induced Modifications in Human Triosephosphate Isomerase: Structural and Functional Repercussions of Specific Mutations
Source: Molecules. 2024 Oct 25;29(21):5047. doi: 10.3390/molecules29215047 (PMC11547674; doi:10.3390/molecules29215047)
Supplement: Supplementary file 1 [file molecules-29-05047-s001.zip › molecules-3213052-supplementary.pdf]

Table S1. Quantification Table of free Cys derivatized by the monomer.

	N° Derivatized Cysteines	
**Enzyme**	**Native**	**(SDS)**
**HsTPI-WT**	**1**	**4**
**C217K**	**0**	**4**
**N16D**	**5**	**0**
**E104D**	**3.9**	**1.1**

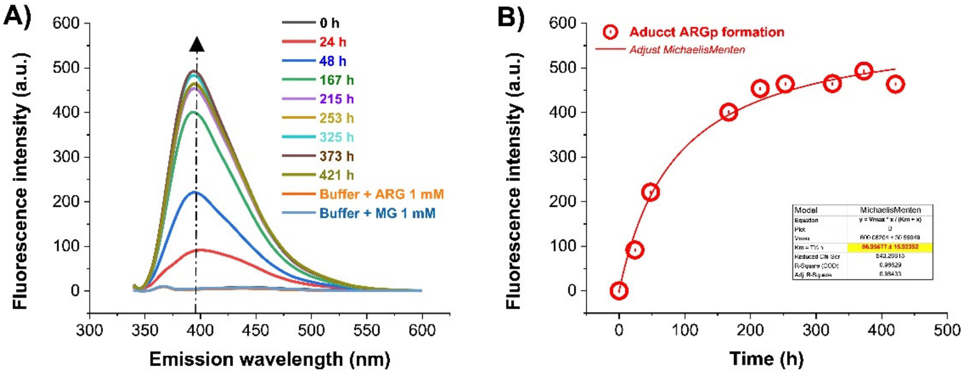

Figure S1. Standard curve of fluorescent ARGp adduct formation. (**A**) The 20 mM Arg-MGO equimolar reaction was included in the TE buffer. Samples at 1:20 dilution were excited at λ exc. 325 nm, the signal of *IFmax* was plotted to be obtained from a scanning of λ em. 340 - 600 nm from 0 to 421 h at 37 °C. (**B**) Michaelis-Menten equation fit of the ARGp formation, plotting the *IFmax* at each time at 395 nm. Curve fitting showed a mean saturation time of 87 h in forming the ARGp adduct. Experimental blanks were subtracted; each spectrum was the mean of three replicate scans.

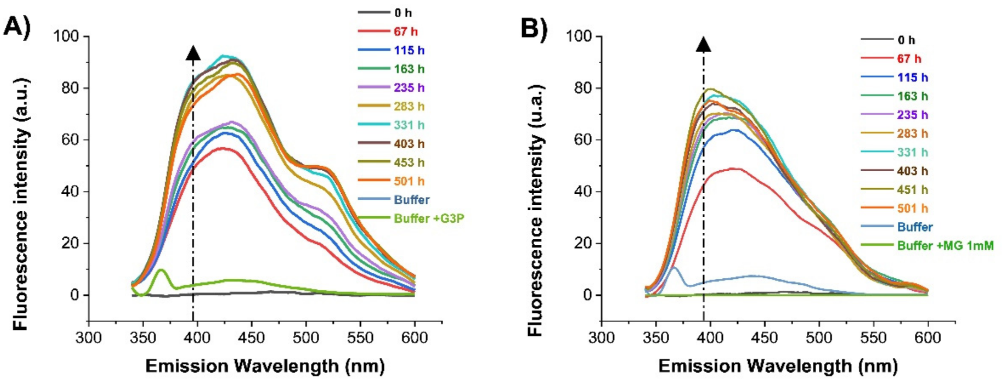

Figure S2. Kinetics of ARGp adduct formation in HsTPI-C217K**.** *IFmax* changes in HsTPI-C217K induced by G3P or MGO were recorded over a 0-500 h incubation in TE buffer at pH 7.4 at 37 °C. Both samples containing 1 mg/mL of HsTPI-C217K plus-G3P 20 mM or plus-MGO 20 mM were incubated. For each time, 1:20 dilutions were made in the same buffer, and spectrofluorometric readings were performed in 600 µL at 25 °C. FI at each time was obtained with 340-600 nm scans after λ exc. at 325 nm. **(A)** C217K plus-G3P. **(B)** C217K plus-MGO. The black arrows show the wavelength signal at 495 nm at which data were collected to construct the graphs (Figures 6A, 7A).

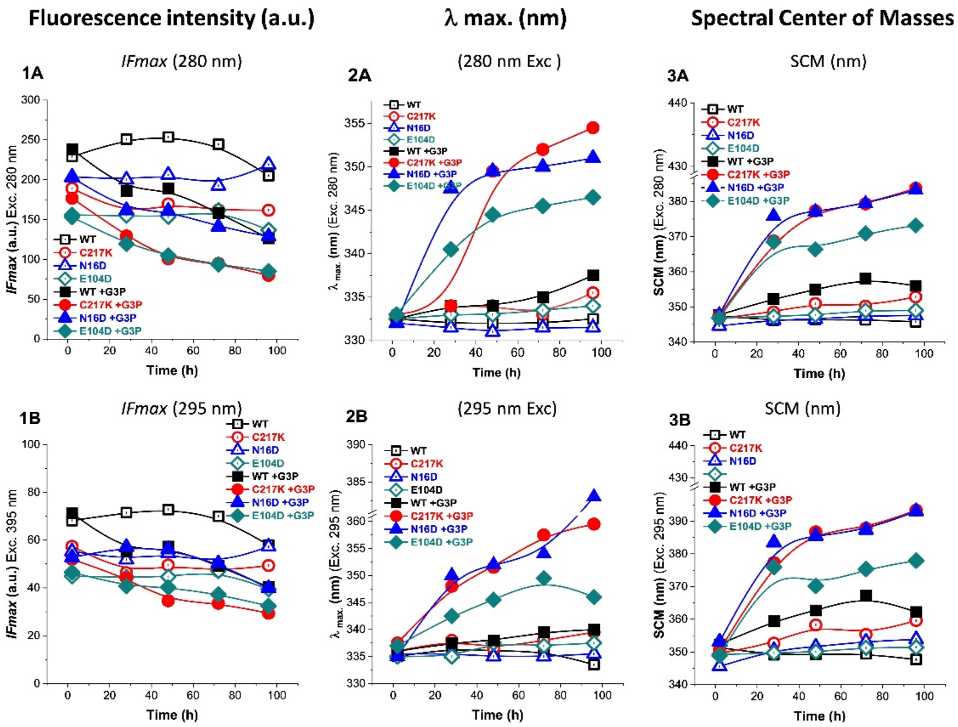

Figure S3. Analysis of structural effects exerted by G3P on TPIs. **(1A-1B)** Fluorescence emission spectra were obtained for the enzyme controls and plus-G3P 2 mM with λ exc at 280 and 295 nm, with readings from 2, 28, 48, 48, 72, and 96 h at 37 °C. The maximum *FI* was substantially modified in the mutants at each time, with strongly decreased plus-G3P relative to the -WT enzyme. **(2A-2B)** the λ max was plotted, red-shift was observed at 280 and 295 nm, and the same behaviour was observed in both λ exc. This inversion of λ max differed among C217K, N16D and E104D, being markedly higher than the WT signal **(3A-3B)**. Spectral centres of mass (SCM) showed the far red shift of three mutants against WT; however, it was higher at 295 nm. Enzymes: (WT, black squares); mutants, (C217K, red circles) (N16D, blue triangles) and (E104D, green diamonds); empty squares show incubation controls; filled squares represent enzymes exposed to 2 mM G3P.

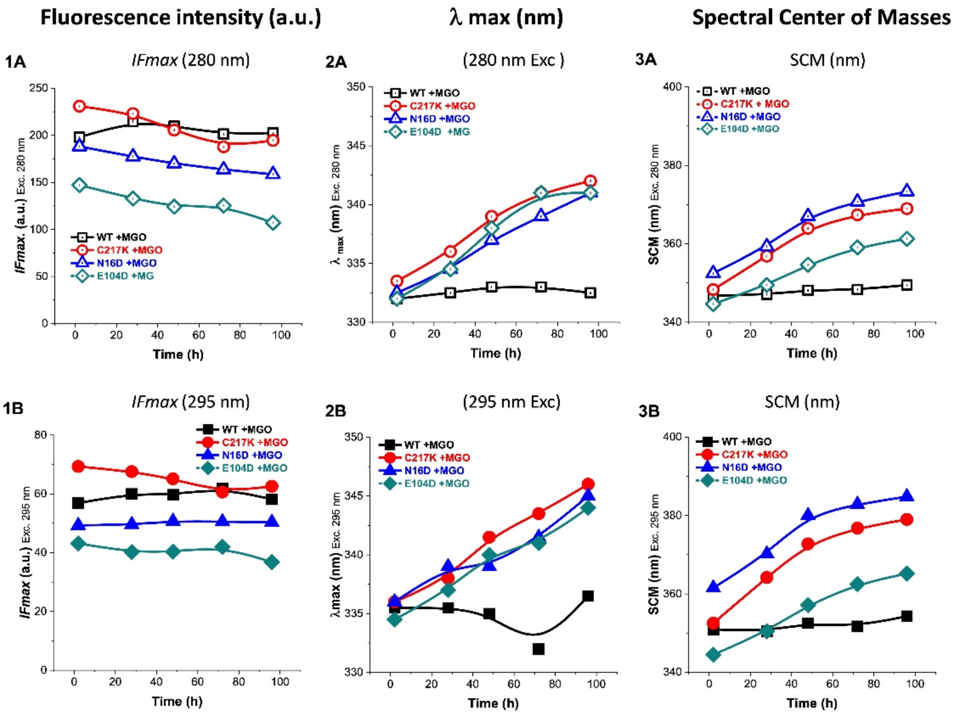

Figure S4. Analysis of structural effect exerted by MGO on TPIs. **(1A-1B)** Fluorescence of enzymes exposed to MGO 1 mM readings from 2, 28, 48, 72, and 96 h at 37 °C at λ exc at 280 and 295 nm. The *IFmax* at exc 280-295 nm was modified in the mutants and decreased to the WT enzyme. **(2A)** The λ max shows a red shift at λ exc—280 nm. The same behaviour was observed at 295 nm. **(2B)** This inversion of the λ max was different in C217K and N16D, being markedly higher than the signal of the WT. **(3A-3B)** SCM showed the far red-shift of both mutants against WT. However, it was higher with excitation at 295 nm. (WT, black squares); mutants, (C217K, red circles) (N16D, blue triangles) and (E104D, green diamonds).

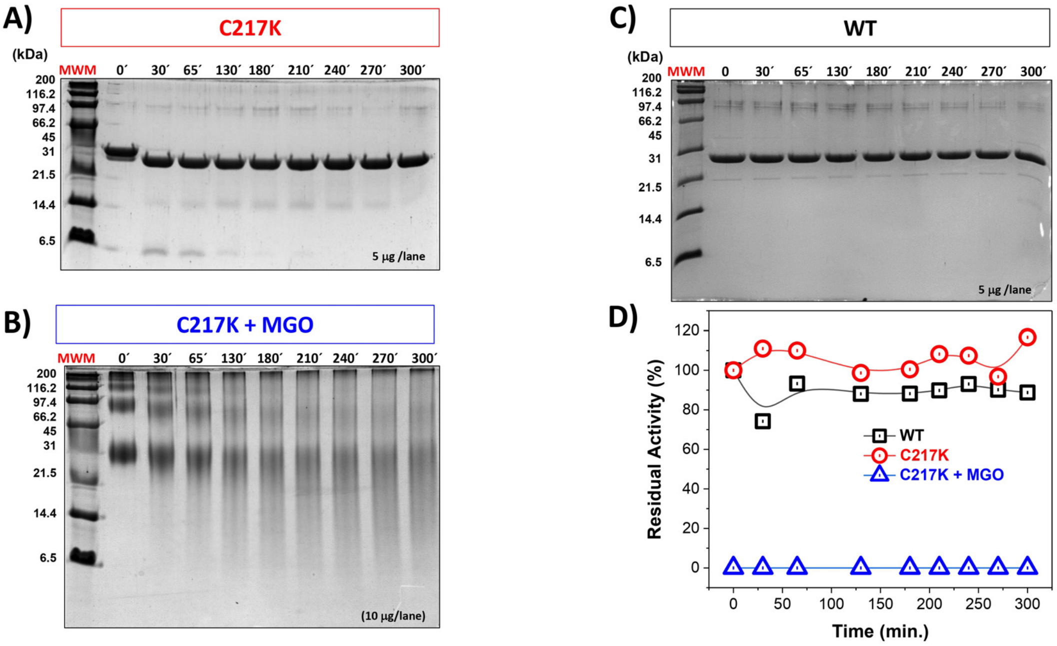

Figure S5. Refractory proteolysis in C217K glycated by MGO. In HsTPI-C217K; **A),** the control lanes were loaded with 5 µg and protein plus MGO 20 mM and glycated by 500 h at 37°C with 10 µg/lane. 16% von Jagow electrophoresis SDS-PAGE was performed to prove the change in the proteolysis pattern. Under the same condition (MGO-free), the digestion pattern is similar in both enzymes WT and C217K. At 500 h, it did not decrease its residual activity against the controls; the enzymes retained their activity (Supplementary Figure S5A and S5C, respectively). However, proteolysis limited of C217K plus MGO showed no residual activity and many resistant aggregates to proteolysis with a sweep on SDS-PAGE observed originating from MGO adducts formation (Supplementary Figure S5C)—a slight disappearance of oligomeric species. Decreasing C217K monomer (27 kDa) increased Mr <monomer fragments. The residual activity was determined after incubation times (0, 30, 65, 130, 180, 210, 240, 270, and 300 min.) at 30 °C. This assay shows that under the same condition, at 5 h of proteolysis, their residual activity did not decrease vs. controls. However, control C217K proved to be catalytically competent and showed a residual activity of 4260.4 µmol·min^{-1}·mg^{-1} that was similar to WT, but C217K incubated plus-MGO did not show residual activity (Supplementary Figure S5D). C217K (red open circles) and C217K plus-MGO (blue open triangles), WT Ctrol (black open squares).

Table S2. Predicted computational binding energies of MGO and Arg ligands to interfacial pocket in TPIs.

PDB ID	MGO docking score (kcal/mol)	Arg docking score (kcal/mol)
**2jk2**	3.2	5.6
**2vom**	3.2	5.9
**4unk**	3.4	6.1
